# Adherence to Mediterranean Diet and Risk of Pancreatic Cancer: Systematic Review and Meta-Analysis

**DOI:** 10.3390/ijerph20032403

**Published:** 2023-01-29

**Authors:** Daniele Nucci, Mariateresa Nardi, Andrea Cinnirella, Emanuela Campagnoli, Manuel Maffeo, Pier Mario Perrone, Viktoriia Shishmintseva, Francesca Maria Grosso, Ambra Castrofino, Silvana Castaldi, Luisa Romanò, Vincenza Gianfredi

**Affiliations:** 1 Nutritional Support Unit, Veneto Institute of Oncology IOV-IRCCS, Via Gattamelata 64, 35128 Padua, Italy; 2 Department of Biomedical Sciences for Health, University of Milan, Via Pascal, 20133 Milan, Italy; 3 Fondazione IRCCS Ca’ Granda Ospedale Maggiore Policlinico, Via Francesco Sforza 35, 20122 Milan, Italy

**Keywords:** pancreatic cancer, Mediterranean diet, diet, systematic review, meta-analysis, pancreas

## Abstract

Pancreatic cancer (PC) represents the 6th cause of cancer death. Although the aetiology of PC is not completely understood, numerous risk factors have been identified in association with this cancer, among them diet. However, little is known about the association between the Mediterranean Diet (MedDiet) and the risk of PC. For this reason, we conducted a systematic review with meta-analysis according to the PRISMA guidelines, searching on three databases (PubMed/MEDLINE, Scopus, and EMBASE). The protocol was registered in PROSPERO. Both fixed and random effect models were performed. The Effect size was reported as a hazard ratio (HR) with a 95% Confidence Interval (CI). A total of eight articles were included. The methodological quality of the included meta-analyses was high. Our results show that a higher adherence to the MedDiet is associated with a lower risk of PC [HR:0.82 (0.76–0.88) *p* < 0.001, based on 1,301,320 subjects]. The results were also confirmed in sensitivity and subgroups analyses (avoidance of potential overlapping effects, type of tools used to assess dietary intake and the diagnosis of PC, prevalence and incidence of PC risk, country where the studies took place, sex, and cancer site). Promoting a higher adherence to the MedDiet could be an effective approach to reduce the risk of PC.

## 1. Introduction

The burden of pancreatic cancer (PC), both in terms of incidence (58.6 cases per million inhabitants, globally), prevalence (49.8 cases per million inhabitants, globally) and mortality (57.7 per million inhabitants, globally), has increased during the past 25 years, currently being the 12th for incidence among all malignancies and the 6th cause of death [1]. Actually, PC is still considered one of the most lethal malignancies, with an overall 5-year survival rate around 5%. The highest burden (both incidence, prevalence and mortality) is registered among subjects aged 80 years or more, even if a gradual increment is observed in subjects aged 30 or more [2].

Although the aetiology of PC is not completely understood, numerous risk factors have been identified in association with this cancer. Among the non-modifiable factors, gender, age and genetic factors have been widely studied. Alcohol and smoking, comorbidities (such as obesity, diabetes, and chronic pancreatitis) and other lifestyle factors, such as diet, have also been studied in association with PC [3,4,5]. For instance, one-third of the deaths from all types of cancer are due to lifestyle and diet [6]. Diet and the risk of PC represents a flourishing area of research, with studies assessing the association between single nutrients, single food or beverage, diets or dietary patterns, and the risk of this cancer [7,8,9,10]. A recent meta-analysis found that a higher intake of dietary fibre is associated with a lower risk of PC [4]. Similarly, a recent umbrella review on the role of diets, dietary patterns, single foods and PC suggested that those characterised by a high consumption of plant-based products, fruits and vegetables, whole grains and nuts, showed a lower risk of PC, with the strongest and most consistent evidence, and with no meta-analyses reporting negative effects [11]. However, the above-mentioned umbrella review failed to find convincing and up-to-date evidence of the association between the Mediterranean diet (MedDiet) and PC.

The Mediterranean diet is characterised by a high intake of vegetables, legumes, fruits and cereals; a high intake of unsaturated fatty acids (mostly in the form of olive oil), but a low intake of saturated fatty acids; a moderately high intake of fish; a low-to-moderate intake of dairy products (mostly cheese or yogurt); a low intake of meat and poultry; and a regular but moderate amount of alcohol [12]. According to several lines of evidence, a higher adherence to the MedDiet is related with a lower risk for cardiovascular disease [13], overall mortality [14], and several forms of cancer [14].

Considering the paucity of evidence about the adherence to the MedDiet and PC risk, and taking into account the high global burden of PC, as well as the importance of identifying and managing the risk factor for PC development, we developed a systematic review and meta-analysis aimed at collecting and collating all of the available evidence on the association between adherence to the MedDiet and PC risk.

## 2. Materials and Methods

### 2.1. Search Strategy and Data Source

This systematic review was developed following the Cochrane Collaboration [15] and the Meta-analysis of Observational Studies in Epidemiology (MOOSE) guidelines [16]. Reporting of the process and the results was conducted based on The Preferred Reporting Items for Systematic Reviews and Meta-Analyses 2020 guidelines (PRISMA) [17]. The structured electronic search was conducted by consulting three different databases: PubMed/MEDLINE, Excerpta Medica Database (EMBASE), and Scopus. The structured search was simultaneously conducted in the three databases on 20 October 2022, combining medical subject headings (MeSH) and free text words. The Boolean operators AND and OR were appropriately and logically combined in order to build the search strategy. Any filter was applied. The search strategy was firstly developed in PubMed and then adapted for the other databases. The keywords identified referred to the Mediterranean diet and synonyms, and to pancreatic cancer and the like. The entire search strategy is available in Table 1. In addition to the three scientific electronic databases, the reference lists of the included studies identified systematic reviews, and meta-analyses were also screened in order to detect any additional related papers. Experts in the field (oncologists) were also consulted. A standardised protocol was developed and shared within the review team and further registered in advance on PROSPERO, the International Prospective Register of Systematic Reviews (ID number: CRD42022367497).

### 2.2. Inclusion/Exclusion Criteria

We only included articles that met the following criteria: (i) written in English; (ii) population: adults ≥ 18 years (both female and male); (iii) interventions or exposures: higher adherence to Mediterranean diet; (iv) comparators/control: lowest or no adherence to Mediterranean diet or adherence to other dietary patterns; (v) outcome: pancreatic cancer risk; however, considering that pancreatic cancer is one of the most lethal malignancies, we will assume any mortality data as data representing incidence. Moreover, any type of original studies (both observational and trial-based) was considered eligible. No restriction on geographical settings was applied. Furthermore, we did not apply any specific Mediterranean diet definition. On the contrary, the mention of this diet in the original manuscript was sufficient for inclusion. In other words, any type of Mediterranean diet scores, as well as, any cut-off used for assessing Mediterranean diet adherence, were considered eligible for inclusion.

Exclusion criteria were: (i) articles not published in English; (ii) people under the age of 18; (iii) interventions or exposures: other diets, dietary patterns, dietary supplementation, single food or food components or micro/macro-nutrients intake; (iv) comparators/control: studies assessing the effect of other diets, dietary patterns, dietary supplementation, single food or food components or micro/macro-nutrients intake; (v) outcome: other outcomes or data combined for pancreatic cancer with other gastrointestinal cancers. Moreover, non-original papers (e.g., review or meta-analysis), articles with no quantitative information or details, no full-text papers (e.g., letters to editor, conference papers, commentary note, expert opinion, abstract), and articles not published as peer-reviewed in international journals were all excluded from the assessment. A detailed description of the inclusion/exclusion criteria is reported in Table 2.

### 2.3. Selection Process and Data Extraction

All retrieved studies were downloaded to EndNote software (EndNote^®^ for Microsoft, Redmond, WA, USA, 2020). Immediately thereafter, duplicates were removed both automatically using EndNote and manually, checked by one review author (VG). Subsequently, a two-step screening process was conducted. Firstly, records were independently screened by two review authors (MM and PMP) based on titles and/or abstracts. Secondly, full-texts were downloaded only for eligible studies and independently assessed by two members of the review team (EC and VS). During both screening steps, any disagreements about the eligibility of papers were solved through discussion among the two review authors; if the disagreement persisted, a third senior reviewer (VG) was involved. Consistently with previous references [18,19], a predefined, standardised spreadsheet, developed in Excel (Microsoft Excel^®^ for Microsoft 365 MSO, Redmond, WA, USA, 2019), was used to extract data from the included articles. The spreadsheet was firstly piloted on three randomly included articles in order to increase the consistency and concordance among the authors [20,21]. The following data was extracted: first author, year of publication, the country where the study took place, study period, study design, number of participants, age and gender, attrition rate, main population characteristics, tool used for dietary assessment, type of MedDiet score, MedDiet range, MedDiet categories, MedDiet items and score system, PC diagnostic tool, type of PC, maximally-adjusted Effect size measures along with the corresponding 95% confidence interval (CI), variables used for adjustment, any possible funds received for conducting the original study, and conflicts of interest declared. The outcome definition was also recorded (this represents a protocol deviation based on a reviewer’s request). The extracted data was used for the assessment of the research quality and evidence synthesis. Two review authors (AC and FMG) independently extracted the data. If any discrepancies arose, they were resolved through discussion between the two; if the disagreement persisted, a third senior author was involved (VG).

### 2.4. Strategy for Data Synthesis

A “flow diagram” charting the number of references at each stage in the review process was produced in line with the PRISMA 2020 guidelines [17]. The quantitative and qualitative results of the literature were summarised in the main body hereof and in descriptive tables. A full report was produced, which contained a narrative overview with a detailed description of the review methodology and findings.

### 2.5. Critical Appraisal

The critical appraisal of all included studies was independently performed by two researchers (AC and ACa), using the Newcastle–Ottawa Scale (NOS) [22]. The NOS is a risk of bias assessment tool for observational studies that assigns a quality score (QS) from zero to nine points based on the risk of bias (zero to the highest risk of bias, nine to the lowest risk of bias). The NOS explores three main domains: (i) study group selection; (ii) comparability; and (iii) ascertainment of exposure and outcomes, respectively, for case–control and cohort studies. Based on these criteria and referring to the standard cut-off previously used [4], the studies were considered of high quality if the NOS score was equal to or greater than 7 points.

### 2.6. Statistical Analysis

The Effect size (ES) was calculated based on the odds ratio (OR), risk ratio (RR), hazard ratio (HR) and mean, and the sample size provided for each study. The ES was reported as the HR with a 95% Confidence Interval (CI). Participants having the highest adherence to the MedDiet were compared to those with the lowest (or no) adherence to the MedDiet. In the current meta-analysis, both fixed and random effect models were used. We choose to follow this approach since the fixed effect model is normally used when studies are considered to be similar. On the contrary, the random effect model is encouraged when the heterogeneity is moderate or high. An I^2^ test was performed to measure the heterogeneity of the included studies. The heterogeneity had four distinct categories: high if the I^2^ values exceeded 75%, moderate if the I^2^ values ranged between 50% and 75%, low for values between 25% and 50%, and no heterogeneity if the values were below 25%. Potential publication bias was assessed by means of both graphical evaluation of the Funnel plot and the Egger’s regression asymmetry test, with statistical significance set at *p* < 0.10 [23]. In case of publication bias, and in order to adjust for it, the trim and fill method, searching missing studies to the right of overall, was performed [24]. Prometa3^®^ software (Internovi, Cesena, Italy) was used to perform the statistical analyses.

### 2.7. Sensitivity and Subgroup Analysis

In the current meta-analysis, several sensitivity and subgroup analyses were conducted. Firstly, we collected studies using the same cohort or study population, and for this reason, we conducted a sensitivity analysis including only those studies with the highest NOS score, or with the largest sample size in the event of the NOS score being equal. This sensitivity analysis was conducted in order to avoid potential overlapping effects. Furthermore, the sensitivity analyses only included studies with validated tools to assess dietary intake; a validated tool to diagnose PC, the study design (prospective and cross-sectional), country where the studies took place, and a QS greater than 7 [25] were also planned.

Subgroup analyses based on the sex and cancer site were also performed to corroborate the results obtained.

All the analyses (main, sensitivity and subgroup analyses) were a priori defined and reported in the registered protocol.

## 3. Results

### 3.1. Literature Search

A total of 245 articles were retrieved, of which 36 were from PubMed/Medline, 125 from EMBASE and 84 from Scopus. After a preliminary screening, 102 articles were excluded because they were duplicates, 93 articles were excluded because they related to other topics, 30 were excluded because they were not original papers (reviews, letters to editor, editorials, protocols, etc.), and 6 articles were removed for being written in a language other than English.

**Figure 1:**
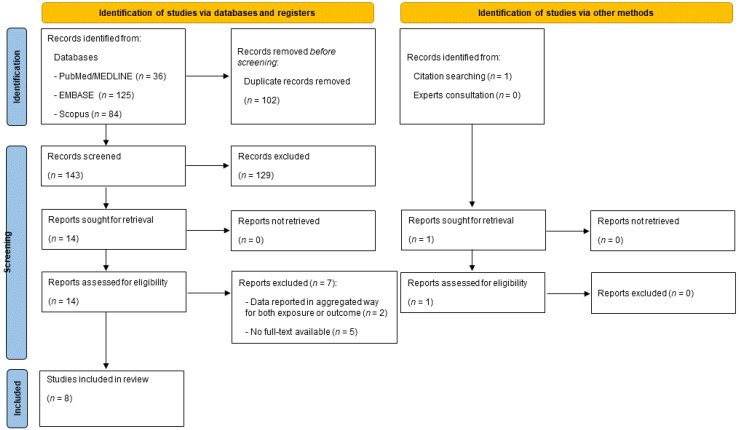
PRISMA flow diagram reporting the selection process.

After the first screening based on the title and abstract, a total of 14 papers were considered eligible. However, seven papers were removed after full text assessment because five papers were conference abstracts (full text was not available) [26,27,28,29,30]; one paper reported results between a combined lifestyle score (including adherence to the MedDiet) and risk of PC [31], and the last paper combined data for PC and other gastrointestinal cancers [32]. A detailed description of the excluded reasons is reported in Supplementary Table S1. At the end of the screening process, a total of eight articles were included in the current systematic review [33,34,35,36,37,38,39,40], of which seven came from the databases and registers screening process [34,35,36,37,38,39,40], and the other was identified from citation searching [33]. Consultation with experts did not add any further eligible studies. The full selection process is depicted in detail in Figure 1.

### 3.2. Main Characteristics of Included Articles

Table 3 and Table 4 describe the main characteristics of the included studies, both qualitative and quantitative data, respectively. Table 3 and Table 4 report studies in alphabetical order.

The first study, assessing the association between adherence to the MedDiet and the risk of PC, was published in 2013 [34]; however, as stated in the registered protocol, we also included a study assessing the association between adherence to the MedDiet and PC mortality. We assumed that since PC is associated with high mortality and rapid death, incidence and mortality can be used interchangeably. The only retrieved study assessing the association between adherence to the MedDiet and PC mortality was a cohort study conducted in Sweden and published in 2012 [40]. However, this study shared the same cohort with another more recent study assessing the association between adherence to the MedDiet and PC risk. For this reason, in order to exclude the potential overlapping effect, we removed it in a sensitivity analysis (see the paragraph below on sensitivity analyses).

Among the eight included studies, almost all (*n* = 6) were conducted in Europe [33,34,37,38,39,40] (Italy, *n* = 2 [34,38], Sweden *n* = 2 [35,39], and The Netherlands *n* = 1 [39]), of which one was a multicentre study, derived from the European Prospective Investigation into Cancer and Nutrition (EPIC) cohort. They included 23 centres scattered across 10 European countries, among them Italy, France, Denmark, Germany, Greece, Spain, Norway, Sweden, United Kingdom and The Netherlands. The remaining two studies were conducted elsewhere, one in the United States of America [35], and the other in Asia (Singapore) [36]. The vast majority of studies were cohort studies (*n* = 6) [33,35,36,37,39,40], with a mean of 15.6 years of follow-up. The remaining two were case-control studies [34,38]. In particular, Bosetti et al. combined data from two different studies; the first one (study period: 1983–1992) was conducted in the province of Milan (northern Italy) on 362 cases of PC and 1.552 controls; whereas the second study (study period: 1992–2008) was conducted in the provinces of Milan and Pordenone (northern Italy) on 326 cases and 652 controls. The matching between the cases and controls was based on age, sex, and study centre, with a matching ratio of 2:1. On the other side, Rosato et al. only presented data coming from the 1992–2008 study period (more details in Table 3). No interventional (trial-based) studies were retrieved.

Referring to the tool used for dietary assessment, all the included studies used a Food Frequency Questionnaire (FFQ) to appraise the frequency and quantity of food intake, although the main differences related to the use of a validated scale and the number of items available in each questionnaire. Regarding the validation process, almost all studies used a validated instrument (*n* = 5), while the rest (*n* = 3) used a mix of structured and validated questionnaires [34,40] or only one structured questionnaire [38]. Among the validated questionnaires, the number of items ranged between 165 [36] and 64 items [33]. On the other hand, referring to the validation process, two authors specified that such process was carried out by comparing the results of the questionnaire through two 24-h recalls [35,36]. Regarding PC diagnosis, all the cohort studies (*n* = 6) used a record linkage with the national cancer databases or death registries. As for the two case-control studies, the information was not specified in one [34], whereas, the other referred to the histological or cytological confirmation, ultrasound and/or tomography [38]. Nevertheless, it should be considered that the two case-control studies shared, at least partially, the same sample, so it could be assumed that the diagnosis was conducted using similar procedures.

Lastly, the results were expressed using two different measures: the Odds Ratio (OR) was adopted by the case-control studies, whereas the Hazard Ratio (HR) was used by the cohort studies.

### 3.3. Characteristics of the Studied Populations

Looking at the cohort studies, the smallest sample size included in a study was 11,268 participants [30], whereas the largest study size was 477,309 participants [37]. Considering the total cases detected, the smallest sample size included 92 cases and 326 cases, among the cohort and case-control studies, respectively. On the contrary, the largest number of cases detected was 3.137 cases, and 688 cases, among the cohort and case-control studies, respectively. The age of the subjects was reported as a range in most of the included studies, while the remainder reported the age as a mean and SD. Generally speaking, the age of participants ranged between 18 and 86 years. Subjects were recruited from the population in all studies, except for the two case-control studies where recruitment was hospital-based (both for cases and for controls). All studies included both males and females; among them, however, one study only reported data separately for males and females, and for this reason, the results were considered separately as two independent studies [39]. All the others reported overall results and separate results for the two sexes. All these results were extracted and set out in Table 4 and used to conduct the subgroup analysis based on sex. Adherence to the MedDiet was reported using different scores. Among them, the alternate Mediterranean diet score was the most frequently used (*n* = 3), followed by the modified Mediterranean diet score (*n* = 2), and the a priori Mediterranean dietary score (*n* = 2). Other scores used were the adapted Mediterranean diet score, the Mediterranean Dietary Pattern Adherence Index, the Mediterranean Adequacy Index, and the non-alcohol relative Mediterranean Diet Score. Most of the included studies assessed the adherence to the MedDiet using multiple scores, as detailed in Table 3.

### 3.4. Mediterranean Diet and Scores Used to Assess Adherence Thereto

Although often described as a lifestyle rather than a diet, the MedDiet is characterised by having a relatively fixed scheme of categories and proportions of different foods, as previously mentioned in the introduction. In order to assess adherence to the MedDiet, all studies adopted the same framework: frequency of food consumption assessed by means of an FFQ on a scale from never to more times a day/week. Total and food-specific daily intake was then calculated for each type of food according to the serving. However, despite the relative uniformity in assessing food consumption, a great variability in operationalizing methods has been observed, so as to account for a total of 9 different scores, which in turn account for different total scores, ranging from 0–7 to 0–44, or even expressing the results as a percentage. In detail, six studies chose to operationalise the MedDiet score using only one method [adapted Mediterranean Diet Score (aMDS) [33], non-alcohol relative Mediterranean Diet (arMED) [37], modified (mMDS) [40], a priori MDS [38], and alternate (aMED) Mediterranean diet scores [35,36]; while the other two calculated the adherence to the MedDiet using more than one method [a priori MDS, Mediterranean Dietary Pattern Index Score (MDPI), and Mediterranean Adequacy Index (MAI)] by Rosato et al. [34], and, lastly, mMDS and aMED by Schulpen et al. [39], who also assessed the adherence to excluding alcohol for the two scores]. Moreover, two studies simultaneously assessed the adherence to different dietary patterns, such as the MedDiet, DASH, or Healthy Eating Index [35,36].

Almost all scores captured the adherence to the MedDiet by assigning one or more points when consumption of a specific food was above the median (when healthy food components were considered) or below it (when the unhealthy food components were considered). Only two scores (MDP and MAI) framed the adherence by summing up the calories yielded by healthy foods and then subtracting those of unhealthy foods (Table 5).

As shown in Table 5, the nine food groups that are most important for a MedDiet (vegetables, fruit, cereals, legumes, fish and fish products, healthy fats, meat and meat products, and dairy products) have been covered by almost all of the MedDiet scores adopted; however, how a specific food has been grouped into a category varied across the studies. For instance, some studies grouped potatoes within the vegetables group, while others did not; likewise with fruits, which in some cases included fresh fruits and nuts, whereas in others, fresh fruits and juices were included. Additionally, some scores split the cereal category into whole grains and refined grains. Lastly, meat and meat products counted poultry as other than red meat, almost every time. Furthermore, consumption of healthy fats was assessed by calculating the Monounsaturated fatty acids (MUFA) + Polyunsaturated fatty acids (PUFA)/saturated fatty acids (SFA) ratio in almost all the scores, except for two [34,37].

Alcohol was accounted for in six out of nine scores; however, the MAI only accounted for red wine consumption. It is worth mentioning that three studies appropriately used a modified version of the score chart in which they removed the alcohol component that could act as a confounder, even under moderate consumption [37,39,40].

### 3.5. Critical Appraisal Results

Regarding the critical assessment, conducted using the NOS scale, the score was generally high, with all studies but one accounting for a score of nine. Only Bodèn et al. totalled a score equal to eight because of an attrition rate (lost to follow-up) of around 60% [23]. A detailed critical appraisal of the included studies is reported item-by-item in Supplementary Table S2. Information regarding fundings was reported in most of the studies (*n* = 5), whereas it was not available in three studies. Moreover, information on conflicts of interest was reported in all but one study; however, only one study declared a potential conflict of interest. All the others stated that the author did not have conflicts of interest (Table 3).

### 3.6. Results of Meta-Analysis

**Figure 2:**
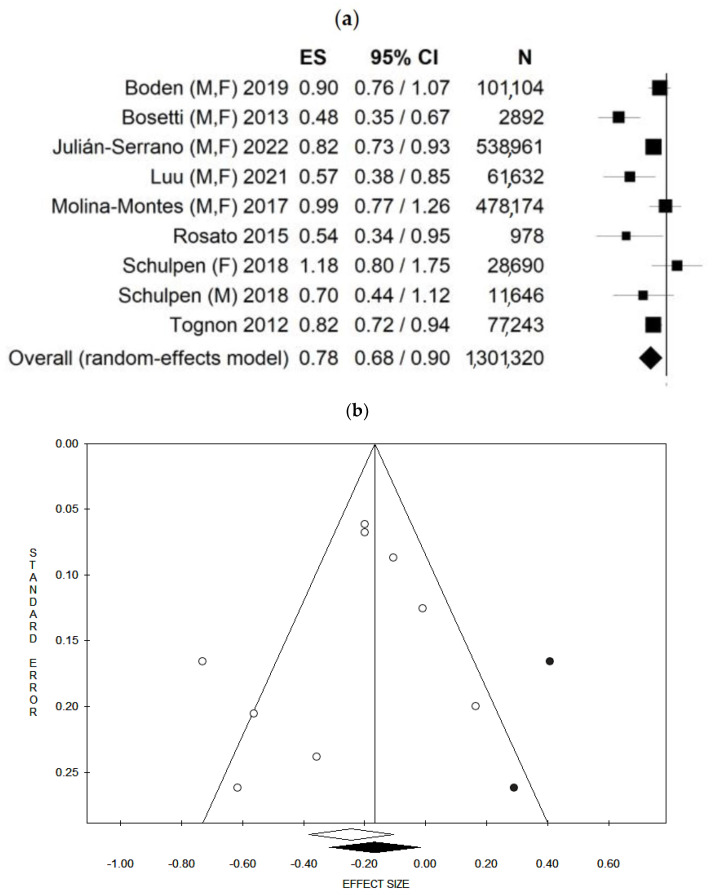
Forest plot (**a**) and Funnel plot (**b**) using the random effect model of the main analysis. The white dots represent the included studies. The white diamond represents the calculated Effect size. The black dots represent the estimated studies after the trim and fill method. The black diamond represents the estimated ES after the trim and fill method. M, F: males and females; M: males, F: females. ES: Effect size, 95% CI: 95% Confidence Interval.

Considering that the studies used several different MedDiet scores, and in order to improve the comparability among the studies, we opted, as much as possible, to pool the risks estimated by using the same MedDiet score. In other words, when more than one score was calculated, we pooled the score most frequently used in the other studies. However, since one study estimated the adherence to the MedDiet using alternate and modified MedDiet scores, which are equally distributed, we decided to perform an additional analysis, alternatively, using the two scores, when available (see below). Moreover, one study reported results separately for males and females, and for this reason it was considered as two independent studies [39]. Based on this, a total of nine data-results were included in the main analysis. Considering all nine data-results, and using the random effect model, the pooled ES was 0.78 [(95% CI = 0.68–0.90), *p*-value = 0.001] based on 1,301,320 participants (Figure 2a) with moderate statistical heterogeneity (df = 8, I^2^ = 65.48, *p*-value = 0.003). Potential publication bias was found by visual assessment of the Funnel plot (Figure 2b) and confirmed by the Egger’s linear regression test (Intercept −1.24, *p*-value = 0.331). After applying the trim and fill method, the estimated Effect sizes did not differ from the main result. Results for both the fixed and random effect models are shown in Table 6.

Moreover, since some studies reported the MedDiet score using more than one score, a secondary analysis, substituting the alternate Mediterranean diet score with the modified Mediterranean diet score, when available, was conducted. Using the random effect model, the pooled ES was 0.77 [(95% CI = 0.68–0.88), *p*-value < 0.001] based on 1,301,320 participants with moderate statistical heterogeneity (df = 8, I^2^ = 60.90, *p*-value = 0.009) (Supplementary Figure S1a). Potential publication bias was found by visual assessment of the Funnel plot (Supplementary Figure S1b) and confirmed by the Egger’s linear regression test (Intercept −1.57, *p*-value = 0.171). However, results for both the fixed and random effect models did not change after applying the trim and fill method. In addition, since one study reported mortality data [40] instead of incidence data, a secondary analysis, only pooling incidence data (excluding the study with mortality data), was conducted. Furthermore, in this case, fixed [HR: 0.81(95% CI = 0.75–0.88); *p* < 0.001] and random effect [HR: 0.77 95% CI = (0.64–0.92); *p* = 0.004] models were used; however, the results did not materially change (this represents a deviation from the protocol since this analysis was added based on a reviewer’s request). The results are reported in Table 6.

### 3.7. Sensitivity Analyses

As mentioned before, two couples of studies share the same sample. In order to estimate the association between adherence to the MedDiet and PC risk without potential overlapping effects, two data-results were excluded [38,40]. Using the random effect model, the pooled ES was 0.79 [(95% CI = 0.66–0.95), *p*-value = 0.001] based on 1,223,009 participants with moderate statistical heterogeneity (df = 6, I^2^ = 70.94, *p*-value = 0.002) (Supplementary Figure S2a). Potential publication bias was found by visual assessment of the Funnel plot (Supplementary Figure S2b) and confirmed by the Egger’s linear regression test (Intercept −1.04, *p*-value = 0.550). After applying the trim and fill method, the estimated ES did not materially change. The results are shown in Table 6.

In order to detect the reason for a moderate-high heterogeneity, a sensitivity analysis only including studies that used validated tools to assess dietary intake, was conducted. In this analysis, four studies were included and, using the random effect model, the pooled ES was 0.84 [(95% CI = 0.70–1.02), *p*-value = 0.084], based on 1,119,103 participants (Supplementary Figure S3a) with moderate statistical heterogeneity (I^2^ = 55.10, *p*-value = 0.063). Potential publication bias was found by visual assessment of the Funnel plot (Supplementary Figure S3b) and confirmed by the Egger’s linear regression test (Intercept −0.04, *p*-value = 0.979). After applying the trim and fill method, the estimated ES did not materially change. The results are shown in Table 6.

A sensitivity analysis, only including studies that used a record linkage to diagnose PC, was conducted. In this analysis, six studies were included, and the pooled ES was 0.86 [(95% CI = 0.78–0.93), *p*-value < 0.001] in the fixed effect model (Supplementary Figure S4a); similarly, using the random effect model, the pooled ES was 0.86 [(95% CI = (0.75–0.99), *p*-value = 0.033] based on 1,220,207 participants with low statistical heterogeneity (I^2^ = 44.64, *p*-value < 0.095). No potential publication bias was found by visual assessment of the Funnel plot (Supplementary Figure S4b), but a borderline statistically significant publication bias was found by the Egger’s linear regression test (Intercept −0.10, *p*-value = 0.947). After applying the trim and fill method, the estimated ES, using the fixed effect model, remained the same; whereas, using the random effect model, the results slightly changed, losing their statistical significance. The results are shown in Table 6.

In order to differentiate between the risk of prevalent and incidental PC, a sensitivity analysis based on study design was conducted. Referring to incidental PC, the results were exactly the same as in the analysis conducted based on record linkage diagnosis, since the studies included were precisely the same. Regarding the prevalent PC, only two case-control studies were susceptible for inclusion. Since it is commonly recognised that at least three studies should be pooled in a meta-analysis, we did not proceed with this analysis.

Moreover, a sensitivity analysis based on the country where the studies took place was also performed. In this case, we combined all the European studies together. Using the random effect model, the HR was 0.82 [(95% CI = 0.62–1.08); *p* = 0.152], with high statistical heterogeneity (I^2^ = 76.57, *p*-value = 0.002). No potential publication bias was found by visual assessment of the Funnel plot (Supplementary Figure S5b), as confirmed by the Egger’s linear regression test (Intercept −1.46, *p*-value = 0.652), both considering the fixed and random effect models.

Despite planning to perform a sensitivity analysis based on quality score, it did not prove possible because all of the assessed and included studies totalled a quality score higher than 7.

### 3.8. Subgroup Analyses

In order to highlight the differences between males and females, a subgroup analysis based on sex was also performed. In particular, when studies only assessing the risk among males were included, and using the random effect model, the HR was 0.89 [(95% CI = 0.78–1.01); *p* = 0.061], with an inverse weak borderline statistical significance, and with high statistical heterogeneity (I^2^ = 76.82, *p*-value = 0.001). A statistically significant publication bias was found after visual inspection of the funnel plot (Supplementary Figure S6b), as confirmed by the Egger’s linear regression test (Intercept −0.10, *p*-value = 0.947). After applying the trim and fill method, the estimated ES using both the fixed and random effect models remained similar. The results are reported in Table 6.

When only studies assessing the risk of PC among females were included, the HR was 0.86 [(95% CI = 0.80–0.93) *p* < 0.001] based on 698,960 (Supplementary Figure S7a), using the fixed effect model, with no statistical heterogeneity (I^2^ = 14.94, *p*-value = 0.318). After applying the random effect model, the results did not materially change. No potential publication bias was found by visual assessment of the Funnel plot (Supplementary Figure S7b), as confirmed by the Egger’s linear regression test (Intercept 0.41, *p*-value = 0.698), both considering the fixed and random effect models (Table 6).

Lastly, since the aetiology of endocrine PC is different compared to the other types of pancreatic carcinomas, a subgroup analysis by cancer type was also performed. In more detail, the subgroup analysis included only four studies that specifically reported an exclusion of subjects with endocrine PC [35,36,38,39]. The current analysis was based on 641.907 subjects with an HR = 0.76 [(95% CI = 0.60–0.96) *p* = 0.021], using the random effect model (Supplementary Figure S8a). In this case, a moderate heterogeneity was found (I^2^ = 56.68, *p*-value = 0.056), with a potential publication bias as demonstrated by visual inspection of the Funnel plot (Supplementary Figure S8b) and as confirmed by the Egger’s linear regression test (Intercept −0.85, *p*-value = 0.556). After applying the trim and fill method, the estimated ES remained virtually unchanged.

## 4. Discussion

### 4.1. Data Interpretation

In the current systematic review with meta-analysis, we estimated the association between the highest adherence to the MedDiet category and the risk of PC. Out of 245 retrieved articles, 14 of them were considered eligible, although at the end of the screening process only 8 articles were included and then analysed. The main grounds for exclusion were because the records retrieved were conference abstracts or because the results were jointly reported for PC and other gastrointestinal cancers. These results suggest that this represents a relatively new area of research, with only eight articles retrieved in total. Moreover, some of them were carried out by the same study group, probably meaning that this topic is explored by selected experts in the field, mainly located in Europe. The main results of this meta-analysis suggest that a higher adherence to the MedDiet is associated with an approximately 20% lower risk of PC (incidence/mortality) out a total sample size of 1,301,320 subjects. In the current meta-analysis, both fixed and random effect models were adopted; however, the results did not differ between the two models, confirming the robustness of our result. Nevertheless, in order to strengthen our results and to assess their robustness, several sensitivity and subgroup analyses were performed. Firstly, since adherence to the MedDiet was assessed using different scores, we pooled the data, alternatively, using the different scores reported within the studies, without impacting on the combined ES. Secondly, because some papers used data from the same sample, we removed any potential overlapping data; however, in this case, too, the results did not materially change. Thirdly, we only included studies that used validated tools, both to assess the exposure (validated FFQ) and the outcome (diagnosis based on record linkage). When only studies using validated FFQ were included, the results did not change; whereas, when only studies using record linkage as a method to define the diagnosis were considered, the risk reduction was slightly lower (15% lower risk of PC, instead of 20% obtained in the main analysis). Nevertheless, a lower level of heterogeneity was detected (I^2^ 46.64%, considered as low heterogeneity). Fourthly, we tried to estimate the strength of the association between an adherence to the MedDiet and both incidental and prevalent PC risk. However, only two studies reported data on prevalent PC risk, and for this reason it was not possible to perform a meta-analysis, whereas, considering the incidental PC risk, results showed a 15% risk reduction, statistically significant considering both fixed and random effect models. Notwithstanding that, it should be considered that longitudinal studies might be more prone to selection bias, especially among PC patients, who might pass away in the short term and maintaining a (long) follow-up could be difficult. In this respect, case-control or cross-sectional studies might also represent a valid study design. However, only two case-control studies, among other things sharing the same sample, were conducted on the topic. In light of this, further case-control or cross-sectional studies are encouraged, particularly to collect data on PC prevalence. Lastly, considering the geographical distribution of the retrieved studies, we performed a sensitivity analysis by grouping studies conducted in Europe. In this case, the results did not change compared to the main analysis (both considering the strengths of the association and heterogeneity) when referring to the fixed effect model, whereas the association became weaker, losing statistical significance, when the random effect model was applied. Moreover, two subgroup analyses were conducted, considering sex and cancer type. Our results showed a risk reduction of approximately 15% among females (with no heterogeneity); however, the reduction was lower when only males were considered (approximately 5%, and only when the fixed effect model was applied). The reason for this different result should be interpreted with caution. Indeed, it could be due to hormonal aspects or because it implies a sex-based different exposure to known or unknown modifiable risk factors. For instance, previous evidence suggests a protective role of female hormones, in particular, hormone replacement therapy (especially an oestrogen-only regimen) was associated with a lower risk of PC [HR = 0.22 (95% CI = 0.05–0.90)]; conversely, a higher age at menarche was significantly associated with PC risk [HR =  1.17 (95% CI 1.04–1.32)] [41]. Nevertheless, it should also be considered that the total sample size based on which we computed the pooled ES was slightly lower among males, compared to females. In this respect, the borderline weak significance association using the random effect model between the MedDiet and PC in males could be purely due to statistical aspects (low statistical power). In light of this, further research is needed in order to better understand whether any differences among the two sexes exist. Lastly, by only focusing on pancreatic adenomas, the strength of the association increased, obtaining a statistically significant 20–25% lower risk of PC.

When comparing our results with previously published meta-analyses on the same topic, we found a statistically significant association between a higher adherence to the MedDiet and lower PC risk, one that was not detected in the two previous meta-analyses. In more detail, Schwingshackl and colleagues published, in 2014, a systematic review and meta-analysis [42], later updated in 2021 [43], assessing the association between adherence to the MedDiet and the risk of several cancers, including PC. In their studies, only two databases were explored (PubMed and Scopus) and a total of four articles were identified [33,34,37,39]. Moreover, they only applied the random effect model and only a sensitivity analysis by study design was conducted. According to their results, adhering to the MedDiet did not modify PC risk [ES = 0.80, (95% CI = 0.60–1.06), with a high level of heterogeneity, I^2^ = 79%. In our view, their results should be considered preliminary, since a fewer number of studies (and consequently a lower total sample size) were considered, minimizing the statistical power of the meta-analysis. Actually, even if Schwingshackl and colleagues did not report the total number of subjects on which the analysis was performed, it is a matter of fact that our sample was larger, considering that we conducted the analysis based on the same articles and on four extra retrieved (and included) studies. However, the results reported by Schwingshackl and colleagues could be seen, at least partially, in line with our results. Actually, despite not reaching statistical significance, the direction of the association comes out in favour of a protective effect of higher adherence to the MedDiet and the risk of PC, as confirmed in our meta-analysis.

### 4.2. Potential Biological Mechanisms

Despite evidence suggesting a pivotal role of the MedDiet in reducing the risk of several non-communicable diseases (NCDs), including cancer [44], the biological mechanism by which beneficial effects occur is still not known. Most likely, the healthy effect of the MedDiet is due to multiple interconnected mechanisms that probably also have synergistic actions. Among the hypothesised biological mechanisms that can explain the anti-cancer effect of the MedDiet, those linked to oxidative stress and chronic inflammation reduction [18,45,46], growth factors regulation, apoptotic cell death induction, and microbiota action, seems to be the most accredited [47].

Moreover, the typical food composition of the MedDiet (prevalently plant-based) seems to lead to significant reductions in the secretion and circulation of insulin, insulin-like growth factor (IGF-1), oestrogen (oestradiol), and testosterone, all of which can stimulate the development and growth of several types of cancer [48]. Moreover, the high intake of omega-3 fatty acids within the MedDiet, seems to exert a cytotoxic effect on cancer cells, inducing apoptosis by different pathways [49].

Lastly, another mechanism through which the MedDiet diet appears to be able to reduce cancer risk is related to the high dietary fibre intake, through direct and indirect mechanisms [4,25,50,51]. In detail, the direct role could be explained by the ability of fibre in the gut to bind carcinogens and remove damaged cells [52]. The indirect effect is mediated by the modification of fibre to short-chain fatty acids (SCFAs) by gut microbiota metabolism. The SCFA production is responsible for the beneficial effect by playing an anticancer role, exerting anti-proliferative, anti-inflammatory and pro-apoptotic effects [53,54].

### 4.3. Strengths and Limitations

Before we generalise, some limitations of the current systematic review and meta-analysis should be considered. Firstly, it represents a secondary study assessing the association between a dietary pattern (MedDiet) and a health outcome (PC risk), so it brings along the intrinsic limitation of studies assessing dietary pattern/intake. Actually, despite the fact that all the included studies used FFQs (most of them validated) to detect the dietary intake, based on which the MedDiet was estimated, it should be borne in mind that diet is extremely hard to measure, given large issues with recall bias, mis-classification and many different sources of measurement error, due for instance, to cultural food behaviours or difficulties in estimating the portion amount within the general population [55]. Moreover, the FFQs used in the original studies had differences among them, increasing the grade of heterogeneity and the certainty of measurement. As a consequence, the potential over or under-estimation obtained in single primary studies is reflected in the secondary evidence, as a systematic review with meta-analysis is. Furthermore, several MedDiet scores are available in the literature. In fact, the MedDiet might be operationalised in different ways, increasing the heterogeneity and the complexity around the topic. Based on the above, it is clear that the association between diet and cancers is complex and requires adjusting by multiple confounding variables. In respect of this, all the included studies largely adjusted the models, in some cases also raising suspicions of a potential over-adjustment. For instance, in one study, the authors simultaneously adjusted for smoking status and smoking pack years [36], potentially underestimating the strength of the association. Moreover, among the included studies, different confounders have been selected and adjusted for in the included studies, potentially impacting the final result. In this case, too, a potential under-estimation of the final results should be considered.

Furthermore, the retrieved studies were mainly conducted among European countries, with low representativeness of Asians and Americans, and no study was conducted among Africans. However, some of them were conducted among north European countries (such as Sweden or The Netherlands) that are traditionally not considered MedDiet-culturally-based countries. In this sense, the adherence to the MedDiet assessed might be more prone to errors considering that most of the foods typically related to the MedDiet are not usually eaten [56]. Further to that, the total level of adherence to the MedDiet could be generally low, facing us with difficulties in detecting differences among the categories.

Another limitation is related to the type of studies retrieved. As mentioned before, only two case-control studies and no trials were found. Indeed, despite the robustness of the methodology applied to conduct the current systematic review with meta-analysis, the understanding of the relationship between the MedDiet and PC is still mainly limited by a lack of randomised controlled trials (the highest level of evidence, albeit limited to primary studies). This may be due to the novelty of the field: actually, all the included studies were published during the last decade.

All the above-mentioned aspects could, at least partially, explain the moderate-to-high heterogeneity detected in the current analysis, and, consequently, the slightly different ESs obtained when using the fixed and random effect models.

Nonetheless, the current systematic review with meta-analysis has certain strengths. First of all, the systematic nature of the review guaranteed the comprehensive approach used to retrieve the available evidence. Actually, three databases were consulted, more than the minimum required by the PRISMA guidelines (at least two databases), alongside a manual check of the listed references and the consultation of experts. Moreover, the review was accompanied by a meta-analysis that allowed an overall risk estimation, adding further robustness and certainty around the topic. Additionally, the conduct and the reporting were documented following international guidelines (Cochrane Collaboration, MOOSE and PRISMA guidelines). The research protocol was registered in advance, increasing the transparency of the entry process. Furthermore, the sample size was particularly large, based on 1,301,320 participants, and, in addition, several sensitivity and subgroup analyses were conducted. Among them, a subgroup analysis by sex was conducted in order to assess gender-specific differences, contributing to the body of evidence related to gender-specific medicine, as recommended by the World Health Organization [57]. Lastly, even if a variety of confounding variables were chosen in the original studies, we combined the data with the highest level of adjustment.

### 4.4. Implications for Public Health Policies and Practice

When considering public health and preventive strategies, this study suggests that adhering to a Mediterranean dietary pattern is associated with a risk reduction of pancreatic cancer, and despite the abovementioned limitations, particularly those related to the dietary assessment methods, our results can be considered reliable thanks to the high sensitivity and subgroup analyses, and the large sample size reached. It is noteworthy that both diet and physical activity have a synergic effect on body composition [58], which in turn seems to be associated with lower cancer risk [59]. Moreover, pooling the maximally-adjusted Effect size measures allowed us to control several potential confounding factors; among them physical activity, smoking, alcohol consumption and energy intake. In fact, previous research shows that having a healthy dietary pattern is commonly associated with having other healthy behaviours as well, such as the regular performance of physical activity or never having been a smoker [35,36]. However, both physical activity and diet are strongly influenced by sociocultural aspects and the environment [60].

Based on the evidence collected so far, and according to the NOURISHING framework of food policies to promote healthy diets, developed by the World Cancer Research Fund (WCRF) [61], investing public money in primary prevention interventions aimed at educating about healthy food choices, and acting on the food environment, such as the regulation of food claims, food advertisements and retail environments, as well as implementing food price discounts, might maximise healthier food choices. These primary prevention initiatives might also have some secondary prevention effects, since healthy diets have been proven to reduce the risk of cancer recurrence [62].

Regarding public policies, our study offers new insights regarding the role of the MedDiet in PC, in relation to gender differences as well. In particular, our data contributes to the creation of a body of knowledge about precision and preventive medicine. This is mainly important if we consider the increasing incident rate and the high mortality rate associated with PC. Moreover, precision and preventive medicine seems to be particularly effective when cancers are taken into account, especially because of the genetic heterogeneity underlying the same histological cancer types.

Last, but not least, the MedDiet does not only represent a healthy food choice, but also a sustainable dietary pattern, and, consequently, promoting higher adherence will improve nutritional intake and reduce the environmental impact, both of which are, in turn, associated with a reduction in disparities.

## 5. Conclusions

To conclude, the findings from the present study might suggest that promoting a higher adherence to the MedDiet may be an effective approach to reduce the risk of PC. The association seems to not change with the type of MedDiet score adopted, nor is it based on geographical distribution, as demonstrated by our sensitivity analyses. However, a higher risk reduction has been observed among women than among men. Nevertheless, these data should be interpretated with caution, since a lower sample size was obtained in the subgroup analysis that only included men. On the basis of the abovementioned limitations, our review underlines the need for future research on this under-explored area.

## Figures and Tables

**Table 1:** Literature search strategy used for each considered database.

Database	Syntax
PubMed/MEDLINE	(((“tumor*” [Title/Abstract] OR “tumour*” [Title/Abstract] OR “cancer*” [Title/Abstract] OR “neoplasm*” [Title/Abstract] OR “malignanc*” [Title/Abstract] OR “neoplastic” [Title/Abstract]) AND (“pancreas” [Title/Abstract] OR “pancreatic” [Title/Abstract])) OR “pancreatic neoplasms” [MeSH Terms]) AND (“diet, mediterranean” [MeSH Terms] OR (“Mediterranean” [Title/Abstract] AND (“diet” [Title/Abstract] OR “diets” [Title/Abstract] OR “dietary pattern” [Title/Abstract] OR “dietary patterns” [Title/Abstract] OR “dietary” [Title/Abstract] OR “dietary adherence” [Title/Abstract] OR “dietary score” [Title/Abstract] OR “dietary scores” [Title/Abstract] OR “dietary index” [Title/Abstract] OR “dietary intervention” [Title/Abstract] OR “dietary behaviour” [Title/Abstract] OR “dietary behavior” [Title/Abstract])))
Scopus	((TITLE-ABS-KEY (pancreatic OR pancreas) AND TITLE-ABS-KEY (neoplasm OR cancer OR tumor OR tumour OR malignanc*))) AND ((TITLE-ABS-KEY (mediterranean) AND TITLE-ABS-KEY (diet OR dietary OR pattern OR intervention OR behavior OR behaviour OR index OR score OR adherence)))
EMBASE	(‘mediterranean diet’/exp OR ((diet* OR dietary OR score* OR intervention* OR ‘behavior’/exp OR behavior OR ‘behaviour’/exp OR behaviour OR ‘index’/exp OR index OR ‘adherence’/exp OR adherence) AND (‘mediterranean’/exp OR mediterranean OR meddiet))) AND (‘pancreas cancer’/exp OR ((‘pancreas’/exp OR pancreas OR pancreatic) AND (neoplasm* OR cancer* OR malignan* OR tumor* OR tumour*)))

**Table 2:** Inclusion/exclusion criteria based on Population, Intervention/Exposure, Comparators/Controls, Outcome, Study design (PI/ECOS) strategy.

Search Strategy	Details
Inclusion criteria	P: adults ≥ 18 years (both female and male) I/E: higher adherence to Mediterranean diet C: lowest or no adherence to Mediterranean diet or adherence to other dietary patterns O: pancreatic cancer risk S: all original study types (both observational and trial-based)
Exclusion criteria	P: people < 18 years old I/E: other diets, dietary patterns, dietary supplementation, single food or food components or micro/macro-nutrients intake C: study assessing the effect of other diets, dietary patterns, dietary supplementation, single food or food components or micro/macro-nutrients intake O: other outcomes or data combined for pancreatic cancer with other gastrointestinal cancers. S: non-original papers, paper without data, articles not published as peer-reviewed in international journals
Language	English
Time filter	No filter (from inception)
Databases	PubMed/Medline; EMBASE, Scopus

**Table 3:** Qualitative characteristics of included studies, reported in alphabetical order.

Author, Year [Ref.]	Country	Study Period	Study Design	Population Characteristics	Tool Used for Dietary Assessment	Mediterranesn Score Used	Diagnostic Assessment	Outcome Definition	Cancer Type	Funds	CoI
Bodèn, 2019 [33]	Sweden	1990–2016; 15 y FU	CO	Male and female from the VIP	three versions of validated FFQs (84-items, 64-items and 66-items)	aMDS	Swedish Cancer Registry	Cases were defined based on ICD-10 codes as the first incident of primary malignancy via annual linkage	All types	yes	yes
Bosetti, 2013 [34]	Italy	1983–1992 first study and 1992–2008 second study	CC	Cases: subjects with pancreatic cancer (without history of previous cancers) admitted to hospitals in the province of Milan Control: subjects admitted to the same network of hospitals as the cases for a wide spectrum of acute, non-neoplastic conditions Matching ratio 2:1, based on age, sex and study centre	structured questionnaire, simplified dietary section (14 selected indicator foods); and validated and reproducible food frequency questionnaire (78 items)	a priori MDS MDPI MAI	n.s.	Incident cases of pancreatic cancer newly admitted to the hospital	n.s.	yes	n.a.
Juliàn-Serrano, 2022 [35]	USA	1995–2011; 15 y FU	CO	NIH–AARP	validated self-administered semiquantitative 124-item FFQ	aMED	Social Security Administration Death Master File	Cases were defined based on ICD-10 codes as the first incident of primary adenocarcinoma via annual linkage	excluding endocrine tumours, sarcomas and lymphomas	n.a.	no
Luu, 2021 [36]	Singapore	1993–2015; 25 y FU	CO	Singapore Chinese Health Study	validated semiquantitative 165-FFQ	aMED	Singapore Cancer Registry and the Singapore Birth and Death Registry	Incident cases of pancreatic cancer were identified via annual linkage	excluding neuroendocrine pancreatic cancer	yes	no
Molina-Montes, 2017 [37]	23 centres in 10 European countries^#^	From ‘90 s to 2004–2008 °; 11 y FU	CO	Male and female from the EPIC cohort	country-specific validated dietary questionnaires (FFQ, diet history, and semiquantitative FFQ)	arMED score	cancer registries and national mortality registries	Incident cancer cases identified via annual linkage	only including exocrine adenocarcinomas	n.a.	no
Rosato, 2015 [38]	Italy	1992–2008;	CC	Cases: subjects with pancreatic cancer (without history of previous cancers) admitted to hospitals in the province of Milan and Pordenone Control: subjects admitted to the same network of hospitals as the cases for a wide spectrum of acute, non-neoplastic conditions Matching ratio 2:1, based on age, sex and study centre	78-item FFQ	a priori MDS	Histological or cytological confirmation (179 patients), ultrasound and/or tomography.	Incident cases of pancreatic cancer newly admitted to the hospital	excluding endocrine pancreatic cancer	yes	no
Schulpen, 2018 [39]	The Netherlands	1986–2006 NLCS and 1993–2014 EPIC-NL; 10 y FU	CO	NLCS and EPIC-NL	validated, self-administered, semiquantitative FFQs (number of items n.s.)	aMED modified MDS (for both scores a non-alcohol score was also estimated)	Netherlands Cancer Registry and nationwide Dutch Pathology Registry	Cases were defined based on ICD-10 codes as the first incident of primary malignancy via annual linkage	excluding endocrine pancreatic cancer	yes	no
Tognon, 2012 [40]	Sweden	1990–2008;18 y FU	CO	VIP	three versions of FFQ (2 × 84-items and 65-items), only one validated	mMDS	Swedish national cause-of-death registry	Deaths were defined based on ICD-10 codes via record linkage	excluding in situ and benign PC	n.a.	no

aMDS: adapted Mediterranean diet score; aMED: alternate Mediterranean score; arMED: non-alcohol relative Mediterranean Diet; CC: case-control study; CO: Cohort study; EPIC: European Prospective Investigation into Cancer and Nutrition cohort; EPIC-NL: European Prospective Investigation into Cancer and Nutrition cohort—Netherlands; FFQ: food frequency questionnaire; FU: Follow-up; y: years; MAI: Mediterranean Adequacy Index; MDPI: Mediterranean Dietary Pattern Adherence Index; MDS: Mediterranean Diet Score; mMDS: modified Mediterranean diet score; n.a.: not available; NIH–AARP: National Institutes of Health (formerly the American Association of Retired Persons) diet and health study; NLCS: Netherlands Cohort Study; n.s.: not specified; PC: pancreatic cancer; USA: United States of America; VIP: Vasterbotten Intervention Programme. ^#^ Italy, France, Denmark, Germany, Greece, Spain, Norway, Sweden, United Kingdom and The Netherlands; ° depending on the study centres; ‘ for unhealthy foods.

**Table 4:** Quantitative characteristics of included studies, reported in alphabetical order.

Author, Year [Ref.]	Total Sample	Attrition *	Sex	Age: Mean and/or Range	MDS Categories	Effect Size (95% CI)	Adjustment	QS/9
Bodèn, 2019 [33]	Ca: 223 Co: 100,881	63%	Ca: F = 107 Co: F = 51,001	40–60 y	Tertiles range n.s.	Total sample: HR = 0.90 (0.76–1.07) Male: HR = 1.01 (0.80–1.28) Female: HR = 0.80 (0.63–1.02)	EI, BMI, smoking, PA, and education	8
Bosetti, 2013 [34]	Ca: 688 Ct: 2204	5%	Ca: F = 285 Co: F = 715	56 (18–84) y	MDPI high ≥ 65.5	Total sample: OR 0.44 (0.27–0.73)	centre, age, sex, year of interview, education, BMI, smoking, alcohol, history of T2D	9
					MAI high ≥ 2.48	Total sample OR 0.68 (0.42–1.11)		
					a priori MDS high ≥ 6 points	Total sample: OR = 0.48 (0.35–0.67) Male: OR = 0.84 (0.78–0.91) Female: OR = 0.87 (0.79–0.97)		
Juliàn-Serrano, 2022 [35]	Ca: 3137 Co: 535,824	5.4%	Ca: F = n.a. Co: F = 220,044	50–71 y	Quintiles, Q1 = 2.5; Q5 = 7.4	Total sample: HR = 0.82 (0.73–0.93) Male: HR = 0.97 (0.95–1.00) Female: HR = 0.76 (0.63–0.92)	age at baseline, sex ^, smoking, BMI, T2D, EI	9
Luu, 2021 [36]	Ca: 311 Co: 61,321	3%	Ca: F = 149 Co: F = n.a.	45–74 y	Quintiles range n.s.	Total sample: HR = 0.57 (0.38–0.85) Male: HR = 0.43 (0.24–0.75) Female: HR = 0.79 (0.45–1.39)	age, sex ^, dialect, year of enrolment, education, smoking, smoking pack-years, coffee drinking, EI, BMI, T2D	9
Molina-Montes, 2017 [37]	Ca: 865 Co: 477,309	2%	Ca: F = 469 Co: F = 335,060	51.5 y	low (0–5 points), medium (6–9 points) high (10–16 points)	Total sample: HR = 0.99 (0.77–1.26) Male: HR = 1.00 (0.68–1.49) Female: HR = 0.99 (0.72–1.37)	EI, BMI, smoking status and intensity, alcohol, T2D	9
Rosato, 2015 [38]	Ca: 326 Ct: 652	0%	Ca: F = 152 Ct: F = 304	63 (34–80) y	low (≤3 points), medium (4–5 points) high (≥6 points)	Total sample: OR = 0.57 (0.34–0.95)	sex, age, study centre, education, BMI, smoking, alcohol, T2D, EI	9
Schulpen, 2018 [39]	Ca: 311 Co: 61,321	14%	Ca: F = 149 Co: F = 28,275	45–74 y	aMED: low (0–3 points), medium (4–5 points) high (6–9 points)	Male: HR = 0.70 (0.44–1.12) Female: HR = 1.18 (0.80–1.75)	age, smoking status, smoking frequency, smoking duration, BMI, EI, alcohol, T2D, family history of pancreatic cancer, education, nonoccupational PA	9
					mMDS: low (0–3 points), medium (4–5 points) high (6–8 points)	Male: HR = 0.66 (0.40–1.10) Female: HR = 0.94 (0.63–1.40)		
Tognon, 2012 [40]	Ca: 92 Co: 77,151		Ca: F = 45 Co: F = 39,605	30–60 y	high > 4 points	Total sample: HR = 0.82 (0.72–0.94) Male: HR = 0.82 (0.68–0.99) Female: HR = 0.83 (0.69–1.00)	age, obesity, smoking status, education, PA	9

* number of subjects lost to follow-up, ^ only in the model for the total sample; BMI: body mass index; Ca: cases; Co: cohort; Ct: controls; MDS: Mediterranean Diet Score; T2D: type 2 diabetes; EI: energy intake, F: female; HR: hazard ratio, MAI: Mediterranean Adequacy Index; MDPI: Mediterranean Dietary Pattern Adherence Index; OR: odds ratio; n.a.: not available; n.s.: not specified; Q: quintiles; QS: quality score; PA: physical activity; y: years.

**Table 5:** Detailed characteristics of the Mediterranean Diet scores adopted in the included studies.

Scale Type	Food Items Included in the Scale												
	Vegetables ^1^	Legumes	Fruit ^2^	Cereals ^3^	Fish ^4^	Healthy ^5^ Fats	Alcohol	Meat and Meat Products	Dairy Products	Added Sugars	Score System	Score Range	Ref.
aMDS	x	x	x	x	x	x	x	x	x	-	1 point if consumption is above */below’ their sex and FFQ-specific median; for alcohol, 1 point if consumption < 50 g/day	0–8	[33]
A priori MDS	x	x	x	x	x	x	x	x	x	-	1 point if consumption is above */below’ their sex and FFQ-specific median	0–9	[34,38]
MDPI	x	x	x	x		x	x	x	x	-	adding up the standardised residuals of the regression of cereals+ fruit+ vegetables+ legumes+ moderate alcohol+ MUFA/SFA ratio on total calories, and subtracting those of milk and meat	0–100%	[34]
MAI	x	x	x	x	x	x	x	x	x	x	dividing the sum of the intake of bread+ cereals+ fruit+ vegetables+ legumes+ potatoes+ fish+ red wine+ vegetable oils as a percentage of total energy by the sum of milk+ cheese+ meat+ eggs+ animal fats and margarines+ sweet beverages+ cakes+ pies+ cookies+ sugar	0–44	[34]
aMED	x	x	x	x	x	x	x	x	-	-	1 point if consumption is above */below’ their study population’s specific median	0–9	[35,36]
arMED	x	x	x	x	x	x	-	x	x	-	A score from 0 to 2 is assigned according to the first, second or third quartile of consumption, respectively (for vegetables, fruits, legumes, fish and cereals); for olive oil, a maximum score of 2 is assigned if consumption is above the median; a score from 0 to 2 is assigned according to the third, second or first quartile of consumption, respectively (for dairy and meat products)	0–16	[37]
mMDS	x	-	x	x	x	x	x	x	x	-	1 point if consumption is above */below’ their sex and FFQ-specific median; for alcohol 1 point if consumption < 50 g/day	0–8	[40]
non-alcohol mMDS	x	-	x	x	x	x	-	x	x	-	1 point if consumption is above */below’ their sex and FFQ-specific median	0–7	[39]
non-alcohol aMED	x	x	x	x	x	x	-	x	-	-	1 point if consumption is above */below’ their sex and FFQ-specific median	0–8	[39]

aMDS: adapted Mediterranean diet score; aMED: alternate Mediterranean score; arMED: non-alcohol relative Mediterranean Diet; MAI: Mediterranean Adequacy Index; MDS: Mediterranean Diet Score; MDPI: Mediterranean Dietary Pattern Adherence Index; mMDS: modified Mediterranean diet score; MUFA: Monounsaturated fatty acids; PUFA: Polyunsaturated fatty acids; SFA: saturated fatty acids; x indicates that the item was included in the scale; - indicates that the item was not part of the scale; * for healthy foods; ‘ for unhealthy foods; ^1^ including potatoes in scores for adapted MDS, modified MDS; including nuts in MDS; ^2^ including fresh juices in scores for adapted MDS, modified MDS, mMEDs, mMEDr; ^3^ including wholegrains in scores aMDS, aMED, mMED, mMEDr. ^4^ including fish products in scores for aMDS, aMED, arMED, mMEDs, mMEDr, ^5^ considered as Ratio of MUFA+PUFA to SFA in scores for aMDS, MDS, MDPI, aMED, mMED, mMEDr, whereas olive oil in scores for armed, MAI.

**Table 6:** Summary statistics of the main, sensitivity and subgroup analyses.

		Summary Statistics				Publication Bias	
Analysis	Studies Included [Ref.]	No. of Participants	df	HR (95% CI); *p*-Value	I^2^; *p*-Value	Intercept’; *p*-Value	Estimated ^a^ ES; *p*-Value
Overall analysis (both male and female) ^	Boden (aMDS), Bosetti (a priori), Juliàn-Serrano (aMED), Luu (aMED), Molina-Montes (arMED), Rosato (a priori), Schulpen (aMED), Tognon (mMED)	1,301,320	8 *	FE: 0.82 (0.76–0.88); *p* < 0.001	65.48%; *p* = 0.003	−1.24, *p* = 0.331	FE: 0.84 (0.79–0.90); *p* < 0.001
				RE: 0.78 (0.68–0.90); *p* = 0.001			RE: 0.84 (0.73–0.97); *p* = 0.017
Overall analysis (both male and female) ^$^	Boden (aMDS), Bosetti (a priori), Juliàn-Serrano (aMED), Luu (aMED), Molina-Montes (arMED), Rosato (a priori), Schulpen (mMED), Tognon (mMED)	1,301,320	8 *	FE: 0.81 (0.76–0.87); *p* < 0.001	60.90%; *p* = 0.009	−1.57; *p* = 0.171	FE: 0.84 (0.78–0.90); *p* < 0.001
				RE: 0.77 (0.68–0.88); *p* < 0.001			RE: 0.83 (0.71–0.96); *p* = 0.014
Excluding mortality data	Boden (aMDS), Bosetti (a priori), Juliàn-Serrano (aMED), Luu (aMED), Molina-Montes (arMED), Rosato (a priori), Schulpen (aMED)	1,224,007	7 *	FE: 0.81 (0.75–0.88); *p* < 0.001	69.79%; *p* = 0.002	−1.36; *p* = 0.351	FE: 0.81 (0.75–0.88); *p* < 0.001
				RE: 0.77 (0.64–0.92); *p* = 0.004			RE: 0.86 (0.79–0.92); *p* < 0.001
Excluding potential overlapping cohorts (both male and female)	Boden (aMDS), Bosetti (a priori), Juliàn-Serrano (aMED), Luu (aMED), Molina-Montes (arMED), Schulpen (aMED)	1,223,009	6 *	FE: 0.82 (0.76–0.89); *p* < 0.001	70.94%; *p* = 0.002	−1.04; *p* = 0.550	FE: 0.87 (0.80–0.94); *p* < 0.001
				RE: 0.79 (0.66–0.95); *p* = 0.001			RE: 0.80 (0.68–0.93); *p* < 0.004
Only studies using validated FFQ	Juliàn-Serrano (aMED), Luu (aMED), Molina-Montes (arMED), Schulpen (aMED)	1,119,103	4 *	FE: 0.84 (0.76–0.93); *p* = 0.001	55.10%; *p* = 0.063	−0.04; *p* = 0.979	FE: 0.86 (0.78–0.95); *p* = 0.002
				RE: 0.84 (0.70–1.02); *p* = 0.084			RE: 0.90 (0.73–1.12); *p* = 0.342
Diagnosis based on record linkage ^+^	Boden (aMDS), Juliàn-Serrano (aMED), Luu (aMED), Molina-Montes (arMED), Schulpen (aMED)	1,220,207	5 *	FE: 0.86 (0.78–0.93); *p* < 0.001	46.64%; *p* = 0.095	−0.10; *p* = 0.947	FE: 0.86 (0.78–0.93); *p* < 0.001
				RE: 0.86 (0.75–0.99); *p* = 0.033			RE: 0.90 (0.77–1.05); *p* = 0.176
Only studies conducted in Europe	Boden (aMDS), Bosetti (a priori), Molina-Montes (arMED), Schulpen (aMED)	622,506	4 *	FE: 0.85 (0.76–0.96); *p* = 0.009	76.57%; *p* = 0.002	−1.46; *p* = 0.652	FE: 0.85 (0.76–0.96); *p* = 0.009
				RE: 0.82 (0.62–1.08); *p*= 0.152			RE: 0.82 (0.62–1.08); *p* = 0.152
**Subgroup analyses**							
Including only male	Boden (aMDS), Bosetti (a priori), Juliàn-Serrano (aMED), Luu (aMED), Molina-Montes (arMED), Schulpen (aMED)	585,430	5	FE: 0.95 (0.93–0.98); *p* < 0.001	76.82%; *p* = 0.001	−1.45; *p* = 0.206	FE: 0.96 (0.93–0.98); *p* < 0.001
				RE: 0.89 (0.78–1.01); *p* = 0.061			RE: 0.91 (0.80–1.05); *p* = 0.197
Including only female	Boden (aMDS), Bosetti (a priori), Juliàn-Serrano (aMED), Luu (aMED), Molina-Montes (arMED), Schulpen (aMED)	698,960	5	FE: 0.86 (0.80–0.93); *p* < 0.001	14.94%; *p* = 0.318	0.41; *p* = 0.698	FE: 0.86 (0.80–0.93); *p* < 0.001
				RE: 0.86 (0.79–0.95); *p* = 0.002			RE: 0.86 (0.79–0.95); *p* = 0.002
Cancer type (excluding endocrine PC)	Juliàn-Serrano (aMED), Luu (aMED), Rosato (a priori), Schulpen (aMED)	641,907	4 *	FE: 0.80 (0.72–0.89); *p* < 0.001	56.68%; *p* = 0.056	−0.85; *p* = 0.556	FE: 0.81 (0.73–0.90); *p* < 0.001
				RE: 0.76 (0.60–0.96); *p* = 0.021			RE: 0.80 (0.64–1.00); *p* = 0.050

aMDS: adapted Mediterranean diet score; aMED: alternate Mediterranean diet score; arMED: non-alcohol relative Mediterranean score; CI: Confident Interval; df: degree of freedom, ES: Effect size; FE: Fixed effect, FQ: food questionnaire; mMED: modified Mediterranean diet score; PC: pancreatic cancer; RE: Random effect. ^ Using, when possible, the alternate Mediterranean diet score. $ Using, when possible, the modified Mediterranean diet score. * In this analysis, Schulpen was considered twice because the results were reported separately for male and female (data-results). ‘ Calculated using the Egger’s linear regression test. ^a^ Estimated using the trim and fill analysis. ^+^ Results of this analysis are the same for the sensitivity analysis including only prospective studies, because the data used is precisely the same.

## Data Availability

Not applicable.

## Supplementary Materials

The following supporting information can be downloaded at: https://www.mdpi.com/article/10.3390/ijerph20032403/s1, Table S1: Detailed reasons for exclusion; Table S2: Item-by-item critical appraisal, evaluated by means of the Newcastle–Ottawa Scale (NOS), of the included studies; Figure S1: Forest plot (a) and Funnel plot (b) using the random effect model of the analysis, substituting the alternate Mediterranean diet score with the modified Mediterranean diet score, when available. The white dots represent the included studies. The white diamond represents the calculated Effect size. The black dots represent the estimated studies after the trim and fill method. The black diamond represents the estimated ES after the trim and fill method. M, F: males and females; M: males, F: females; Figure S2: Forest plot (a) and Funnel plot (b) using the random effect model of the sensitivity analysis excluding the potential overlapping effect. Studies are reported in alphabetical order. The white dots represent the included studies. The white diamond represents the calculated Effect size. The black dot represents the estimated studies after the trim and fill method. The black diamond represents the estimated ES after the trim and fill method. M, F: males and females; M: males, F: females.; Figure S3. Forest plot (a) and Funnel plot (b) using the random effect model of the sensitivity analysis including only studies that used validated tools to assess dietary intake. Studies are reported in alphabetical order. The white dots represent the included studies. The white diamond represents the calculated Effect size. The black dot represents the estimated studies after the trim and fill method. The black diamond represents the estimated ES after the trim and fill method. M, F: males and females; M: males, F: females; Figure S4: Forest plot (a) and Funnel plot (b) using the fixed effect model of the sensitivity analysis, including only studies that used record linkage to diagnosis pancreatic cancer. Studies are reported in alphabetical order. The white dots represent the included studies. The white diamond represents the calculated Effect size. M, F: males and females; M: males, F: females; Figure S5: Forest plot (a) and Funnel plot (b) using the random effect model of the sensitivity analysis, including only studies conducted in Europe. Studies are reported in alphabetical order. The white dots represent the included studies. The white diamond represents the calculated Effect size. M, F: males and females; M: males, F: females; Figure S6: Forest plot (a) and Funnel plot (b) using the random effect model of the subgroup analysis including only males. Studies are reported in alphabetical order. The white dots represent the included studies. The white diamond represents the calculated Effect size. The black dots represent the estimated studies after the trim and fill method. The black diamond represents the estimated ES after the trim and fill method. M: males; Figure S7: Forest plot (a) and Funnel plot (b) using the fixed effect model of the subgroup analysis including only females. Studies are reported in alphabetical order. The white dots represent the included studies. The white diamond represents the calculated Effect size. F: females; Figure S8. Forest plot (a) and Funnel plot (b) using the random effect model of the subgroup analysis based on cancer type (endocrine pancreatic cancers were excluded). Studies are reported in alphabetical order. The white dots represent the included studies. The white diamond represents the calculated Effect size. The black dot represents the estimated studies after the trim and fill method. The black diamond represents the estimated ES after the trim and fill method. M, F: males and females; M: males, F: females.
