# Supplementary material for: Adherence to Mediterranean Diet and Risk of Pancreatic Cancer: Systematic Review and Meta-Analysis
Source: Int J Environ Res Public Health. 2023 Jan 29;20(3):2403. doi: 10.3390/ijerph20032403 (PMC9915542; doi:10.3390/ijerph20032403)

Supplementary Table S1. Detailed reasons of exclusion

References	n	Reasons of exclusion
Steel, 2022; Luu, 2021; Luu 2021, Julián-Serrano, 2021; Schulpen, 2018	5	Conference abstract without full-text
Jiao, 2009	1	Results reported combining MedDiet with other lifestyle factors
Barrea, 2018	1	Results reported combining PC with other gastrointestinal cancer

Supplementary Table S2. Item-by-item critical appraisal, evaluated by means of the New-Castle Ottawa Scale (NOS), of included studies, stratified by study design and reported in alphabetical order.

NEWCASTLE - OTTAWA QUALITY ASSESSMENT SCALE - COHORT STUDIES

STUDY	SELECTION				COMPARABILITY	OUTCOME			
Author, year	Representativeness of the exposed cohort (Maximum: *)	Selection of the non exposed cohort (Maximum: *)	Ascertainment of exposure (Maximum: *)	Demonstration that outcome of interest was not present at start of study (Maximum: *)	Comparability of cohorts on the basis of the design or analysis (Maximum: **)	Assessment of outcome (Maximum: *)	Was follow-up long enough for outcomes to occur (Maximum: *)	Adequacy of follow up of cohorts (Maximum: *)	TOTAL SCORE
Bodén, 2019	*	*	*	*	**	*	*		8
Julián-Serrano, 2022	*	*	*	*	**	*	*	*	9
Luu, 2021	*	*	*	*	**	*	*	*	9
Molina-Montes, 2017	*	*	*	*	**	*	*	*	9
Schulpen, 2018	*	*	*	*	**	*	*	*	9
Tognon, 2012	*	*	*	*	**	*	*	*	9

NEWCASTLE - OTTAWA QUALITY ASSESSMENT SCALE - CASE CONTROL STUDIES

STUDY	SELECTION				COMPARABILITY	OUTCOME			
Author, year	Is the case definition adequate? (Maximum: *)	Representativeness of the cases (Maximum: *)	Selection of Controls (Maximum: *)	Definition of Controls (Maximum: *)	Comparability of cases and controls on the basis of the design or analysis (Maximum: **)	Ascertainment of exposure (Maximum: *)	Same method of ascertainment for cases and controls (Maximum: *)	Non-Response rate (Maximum: *)	TOTAL SCORE
Bosetti, 2013	*	*	*	*	**	*	*	*	9
Rosato, 2015	*	*	*	*	**	*	*	*	9

Supplementary Figure S1. Forest plot (a) and funnel plot (b) using random effect model, of the analysis substituting alternate Mediterranean diet score with modified Mediterranean diet score, when available. The white dots represent the included studies. The white diamond represents the calculates Effect size. The black dots represent the estimated studies after the trim and fill method. The black diamond represents the estimated ES after the trim and fill method. M, F: males and females; M: males, F: females.

a)

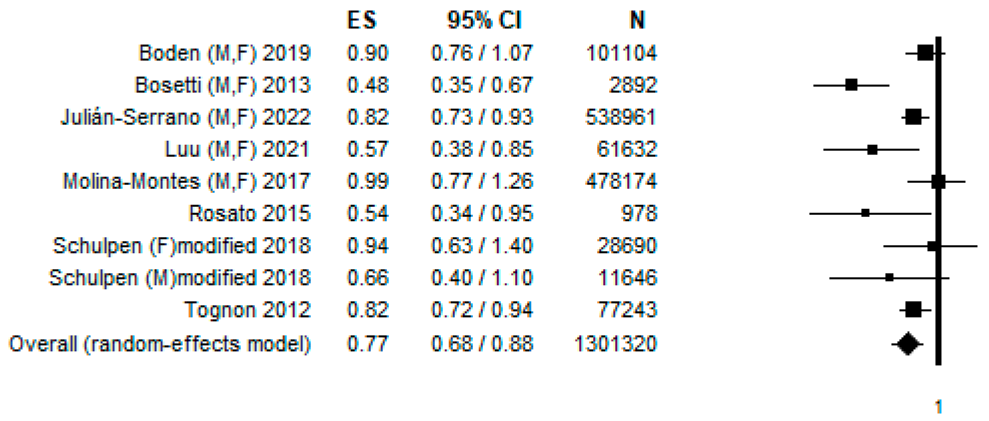

b)

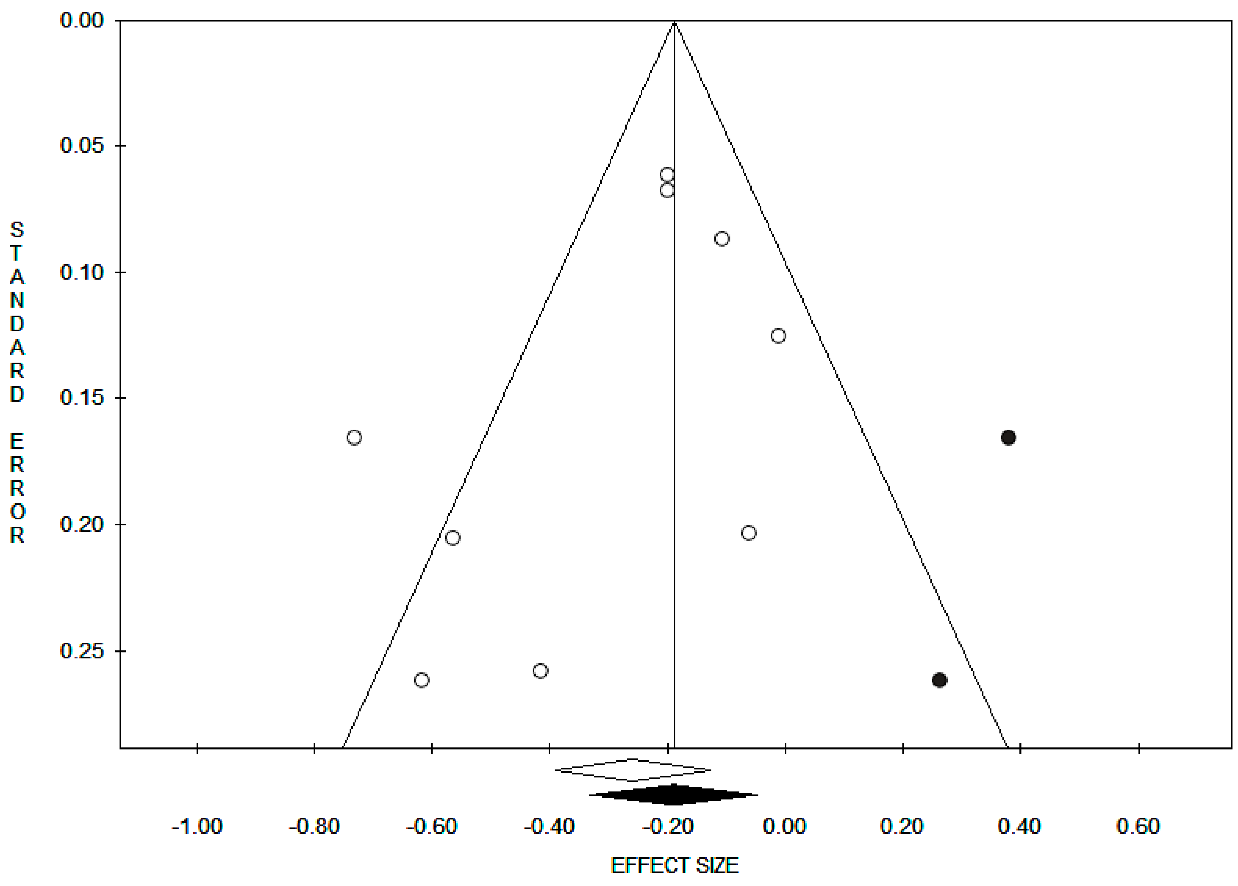

Supplementary Figure S2. Forest plot (a) and funnel plot (b) using random effect model, of the sensitivity analysis excluding potential overlapping effect. Studies are reported in alphabetical order. The white dots represent the included studies. The white diamond represents the calculates Effect size. The black dot represents the estimated studies after the trim and fill method. The black diamond represents the estimated ES after the trim and fill method. M, F: males and females; M: males, F: females.

a)

	ES	95% CI	N
Boden (M,F) 2019	0.90	0.76 / 1.07	101104
Bosetti (M,F) 2013	0.48	0.35 / 0.67	2892
Julián-Serrano (M,F) 2022	0.82	0.73 / 0.93	538961
Luu (M,F) 2021	0.57	0.38 / 0.85	61632
Molina-Montes (M,F) 2017	0.99	0.77 / 1.26	478174
Schulpen (F) 2018	1.18	0.80 / 1.75	28690
Schulpen (M) 2018	0.70	0.44 / 1.12	11646
Overall (random-effects model)	0.79	0.66 / 0.95	1223099

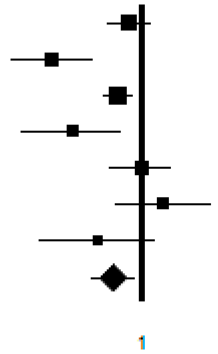

b)

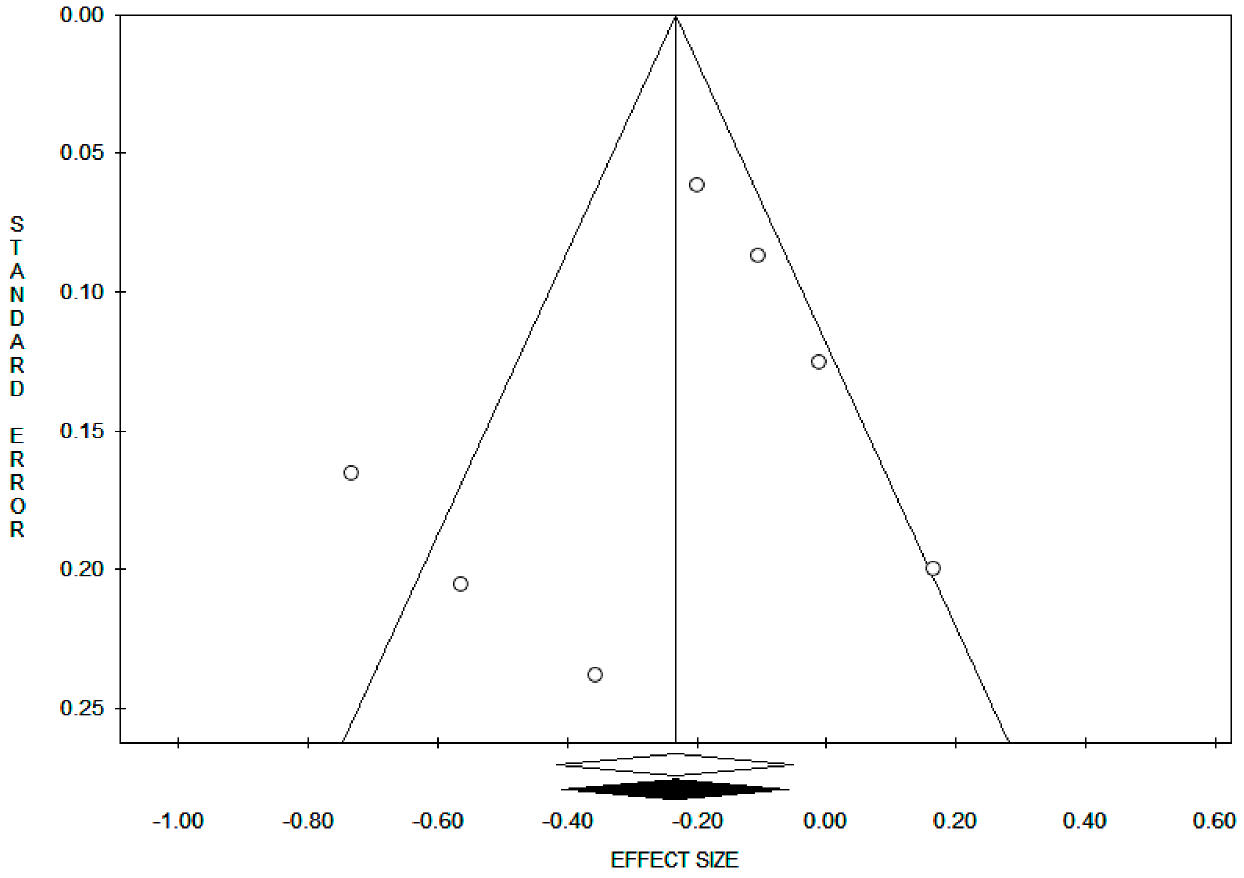

Supplementary Figure S3. Forest plot (a) and funnel plot (b) using random effect model, of the sensitivity analysis only including studies that used validated tools to assess dietary intake. Studies are reported in alphabetical order. The white dots represent the included studies. The white diamond represents the calculates Effect size. The black dot represents the estimated studies after the trim and fill method. The black diamond represents the estimated ES after the trim and fill method. M, F: males and females; M: males, F: females.

a)

	ES	95% CI	N
Julián-Serrano (M,F) 2022	0.82	0.73 / 0.93	538961
Luu (M,F) 2021	0.57	0.38 / 0.85	61632
Molina-Montes (M,F) 2017	0.99	0.77 / 1.26	478174
Schulpen (F) 2018	1.18	0.80 / 1.75	28690
Schulpen (M) 2018	0.70	0.44 / 1.12	11646
Overall (random-effects model)	0.84	0.70 / 1.02	1119103

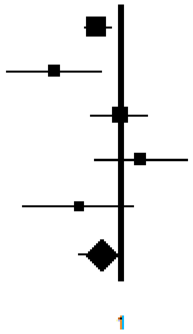

b)

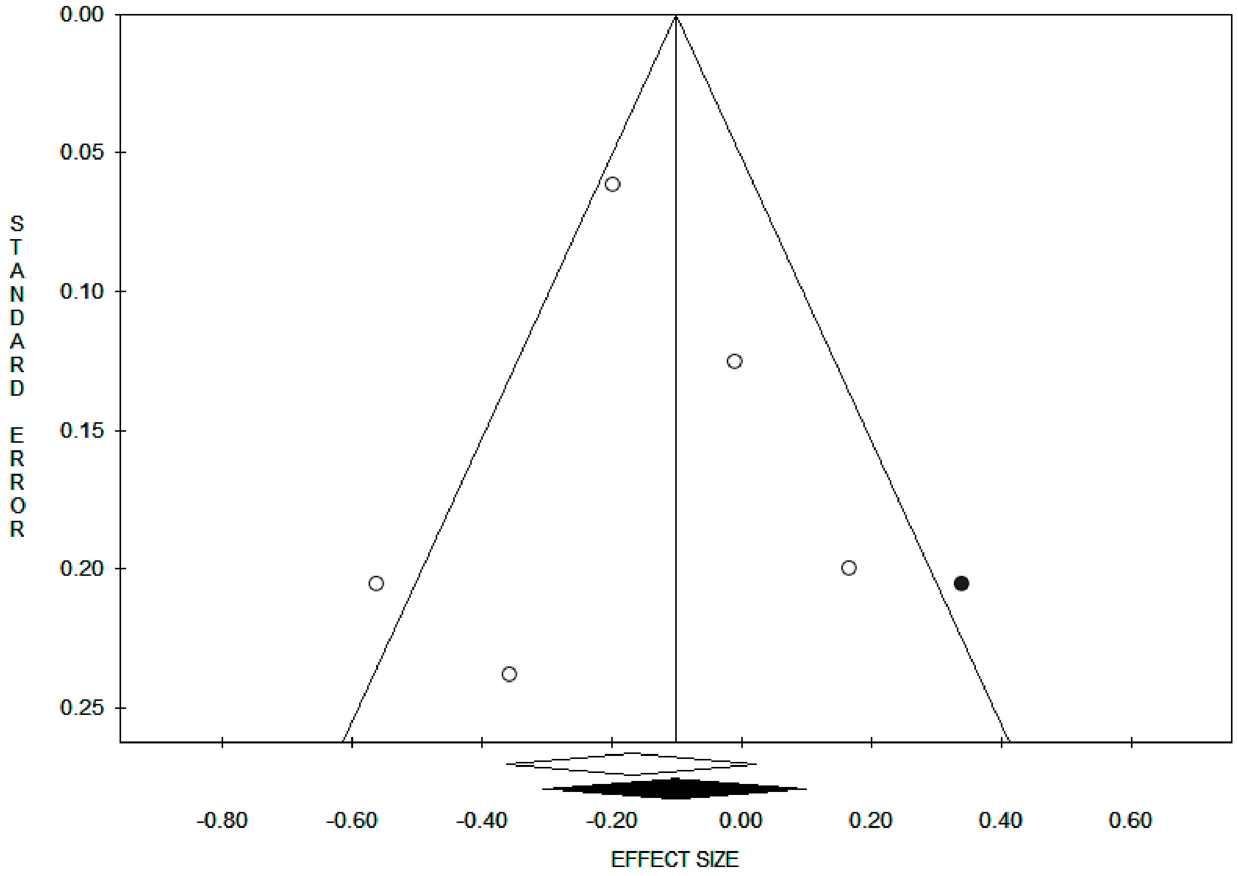

Supplementary Figure S4. Forest plot (a) and funnel plot (b) using fixed effect model, of the sensitivity analysis only including studies that used record linkage to diagnosis pancreatic cancer. Studies are reported in alphabetical order. The white dots represent the included studies. The white diamond represents the calculates Effect size. M, F: males and females; M: males, F: females.

a)

	ES	95% CI
Boden (M,F) 2019	0.90	0.76 / 1.07
Julián-Serrano (M,F) 2022	0.82	0.73 / 0.93
Luu (M,F) 2021	0.57	0.38 / 0.85
Molina-Montes (M,F) 2017	0.99	0.77 / 1.26
Schulpen (F) 2018	1.18	0.80 / 1.75
Schulpen (M) 2018	0.70	0.44 / 1.12
Overall (fixed-effect model)	0.86	0.78 / 0.93

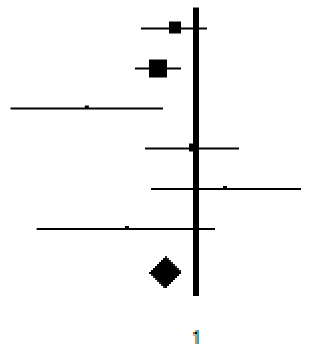

b)

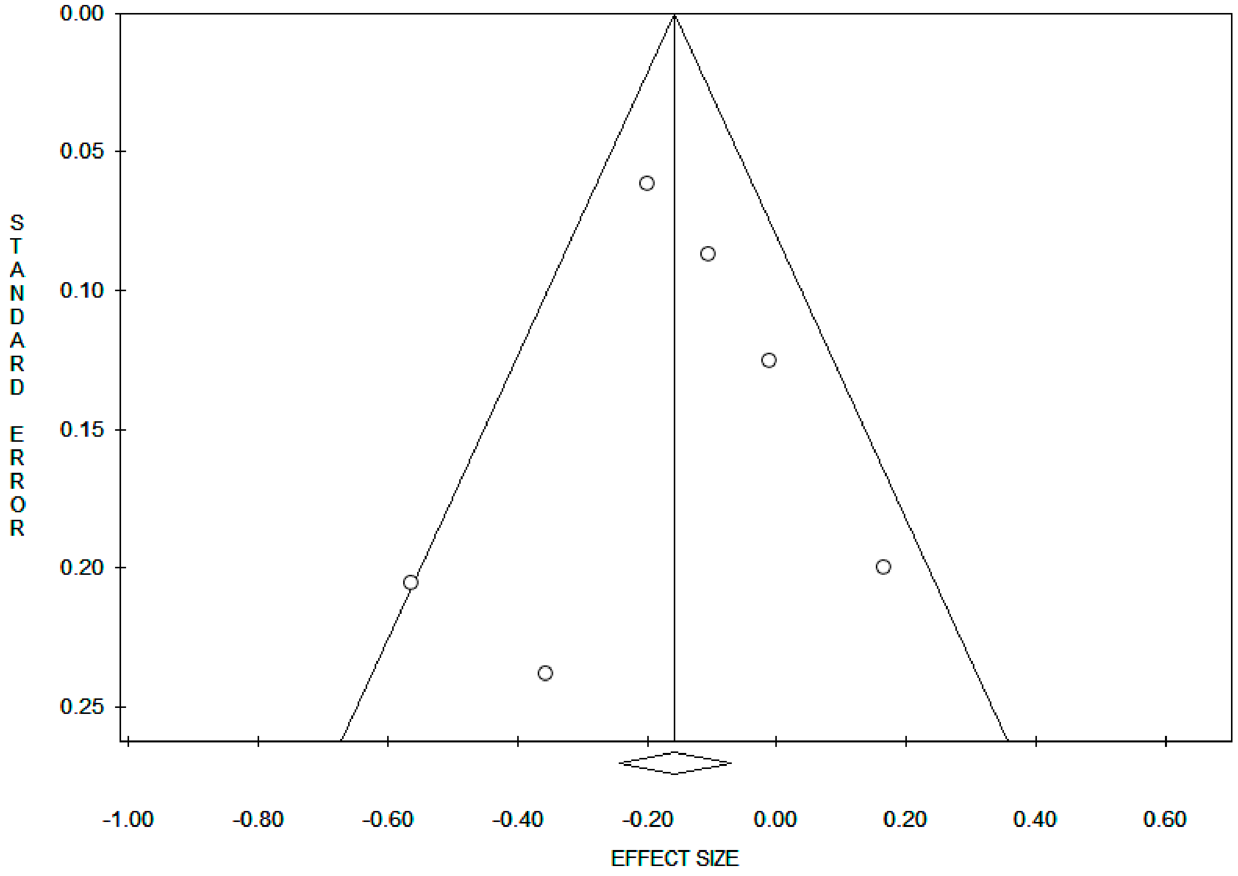

Supplementary Figure S5. Forest plot (a) and funnel plot (b) using random effect model, of the sensitivity analysis only including studies conducted in Europe. Studies are reported in alphabetical order. The white dots represent the included studies. The white diamond represents the calculates Effect size. M, F: males and females; M: males, F: females.

a)

	ES	95% CI	N
Boden (M,F) 2019	0.90	0.76 / 1.07	101104
Bosetti (M,F) 2013	0.48	0.35 / 0.67	2892
Molina-Montes (M,F) 2017	0.99	0.77 / 1.26	478174
Schulpen (F) 2018	1.18	0.80 / 1.75	28690
Schulpen (M) 2018	0.70	0.44 / 1.12	11646
Overall (random-effects model)	0.82	0.62 / 1.08	622506

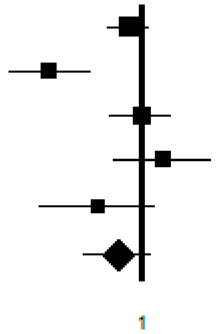

b)

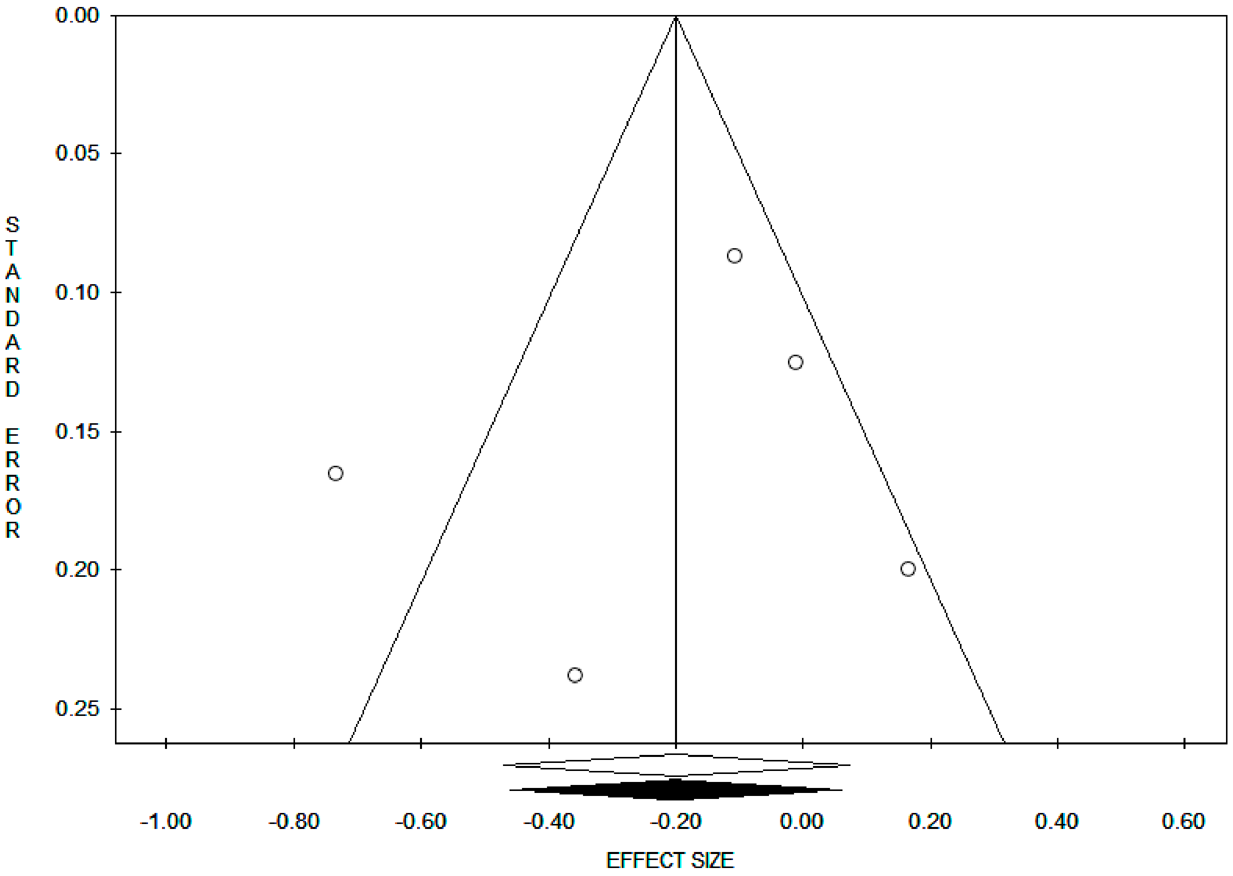

Supplementary Figure S6. Forest plot (a) and funnel plot (b) using random effect model, of the subgroup analysis only including males. Studies are reported in alphabetical order. The white dots represent the included studies. The white diamond represents the calculates Effect size. The black dots represent the estimated studies after the trim and fill method. The black diamond represents the estimated ES after the trim and fill method. M: males.

a)

	ES	95% CI	N
Boden (M) 2019	1.01	0.80 / 1.28	49996
Bosetti (M) 2013	0.84	0.78 / 0.91	1892
Julián-Serrano (M) 2022	0.97	0.95 / 1.00	317768
Luu (M) 2021	0.43	0.24 / 0.75	61483
Molina-Montes (M) 2017	1.00	0.68 / 1.49	142645
Schulpen (M) 2018	0.70	0.44 / 1.12	11646
Overall (random-effects model)	0.89	0.78 / 1.01	585430

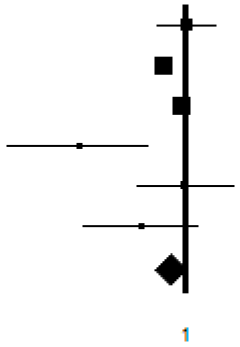

b)

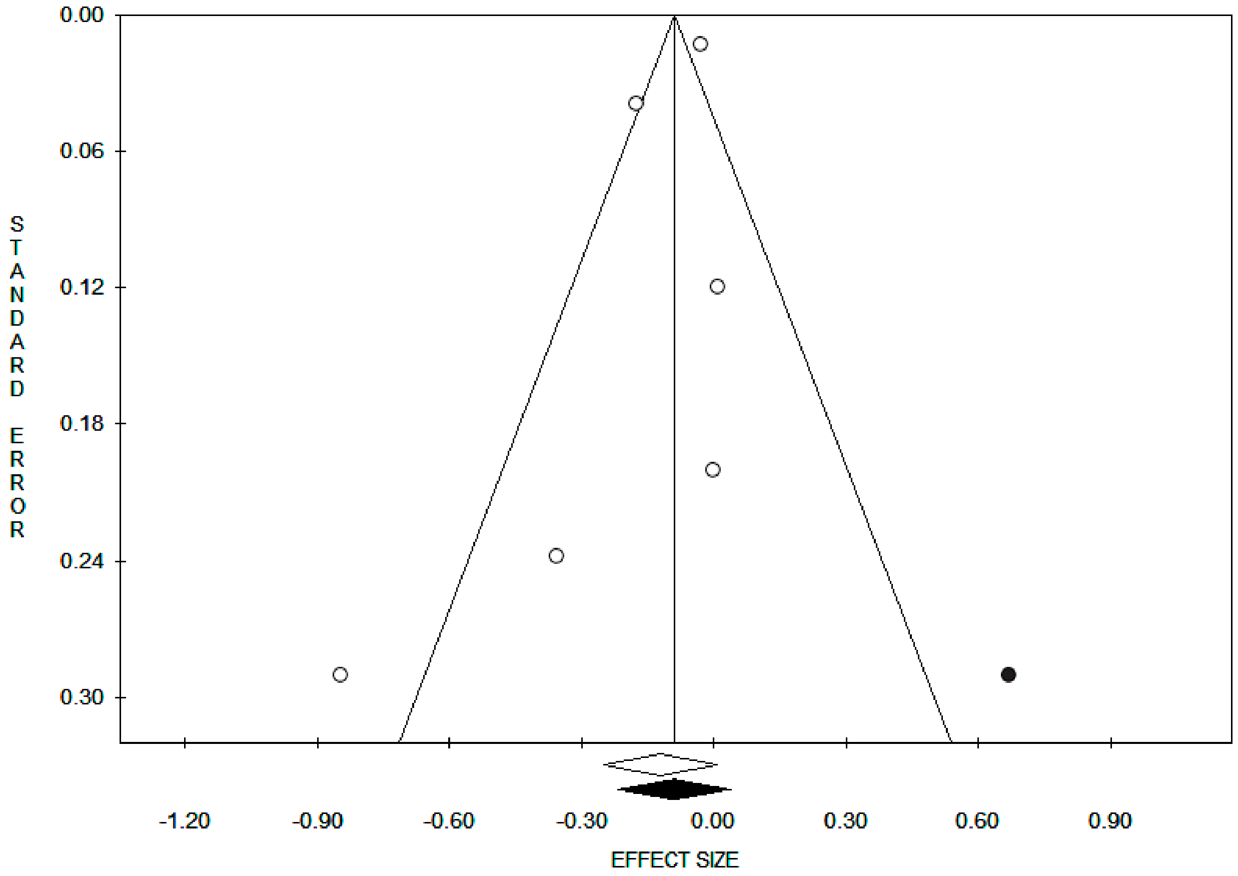

Supplementary Figure S7. Forest plot (a) and funnel plot (b) using fixed effect model, of the subgroup analysis only including females. Studies are reported in alphabetical order. The white dots represent the included studies. The white diamond represents the calculates Effect size. F: females.

a)

	ES	95% CI
Boden (F) 2019	0.80	0.63 / 1.02
Bosetti (F) 2013	0.87	0.79 / 0.97
Julián-Serrano (F) 2022	0.76	0.63 / 0.92
Luu (F) 2021	0.79	0.45 / 1.39
Molina-Montes (F) 2017	0.99	0.77 / 1.26
Schulpen (F) 2018	1.18	0.80 / 1.75
Overall (fixed-effect model)	0.86	0.80 / 0.93

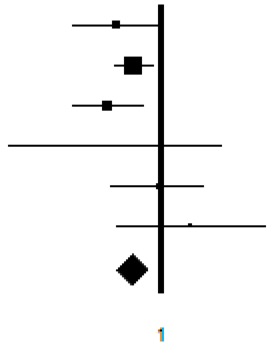

b)

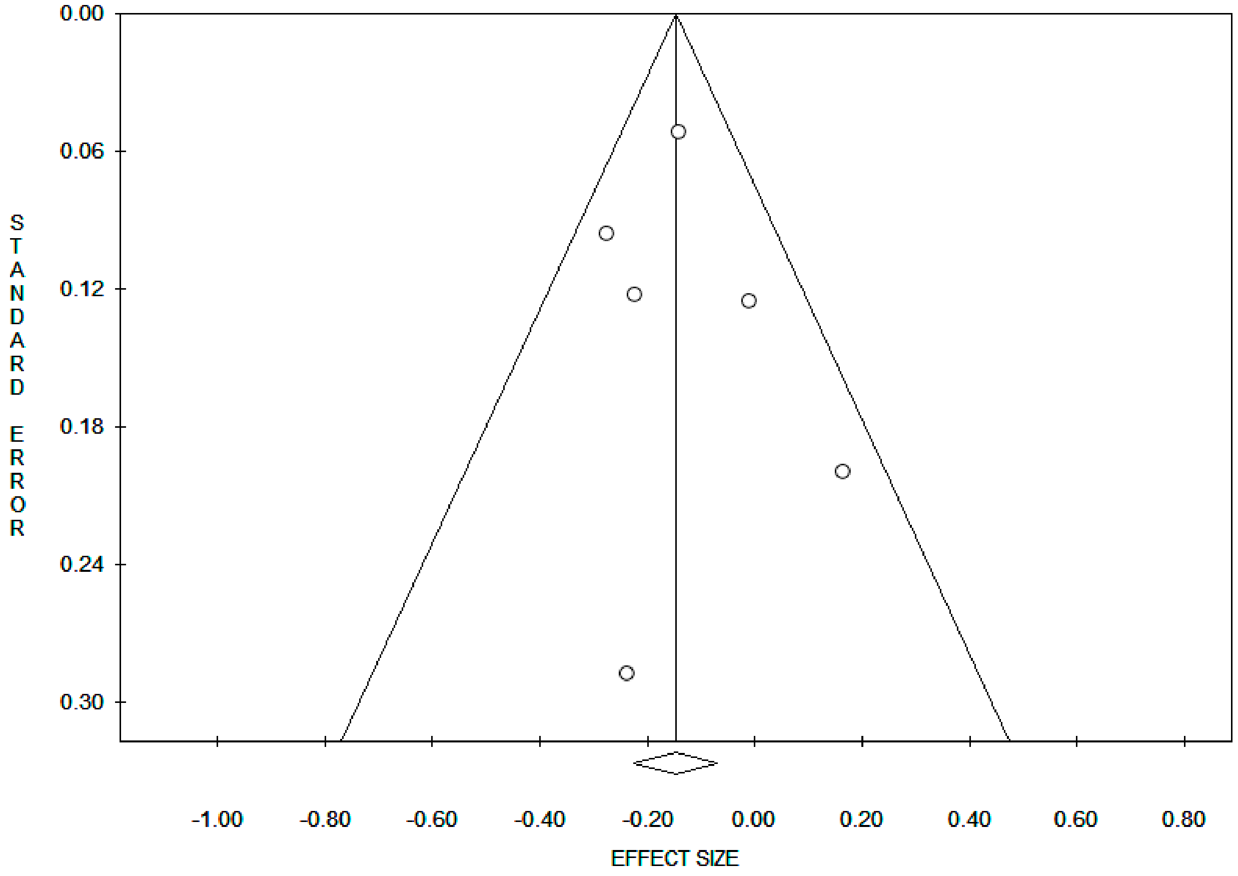

Supplementary Figure S8. Forest plot (a) and funnel plot (b) using random effect model, of the subgroup analysis based on cancer type (endocrine pancreatic cancers were excluded). Studies are reported in alphabetical order. The white dots represent the included studies. The white diamond represents the calculates Effect size. The black dot represents the estimated study after the trim and fill method. The black diamond represents the estimated ES after the trim and fill method. M, F: males and females; M: males, F: females.

a)

	ES	95% CI	N
Julián-Serrano (M,F) 2022	0.82	0.73 / 0.93	538961
Luu (M,F) 2021	0.57	0.38 / 0.85	61632
Rosato 2015	0.54	0.34 / 0.95	978
Schulpen (F) 2018	1.18	0.80 / 1.75	28690
Schulpen (M) 2018	0.70	0.44 / 1.12	11646
Overall (random-effects model)	0.76	0.60 / 0.96	641907

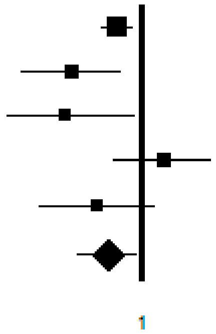

b)

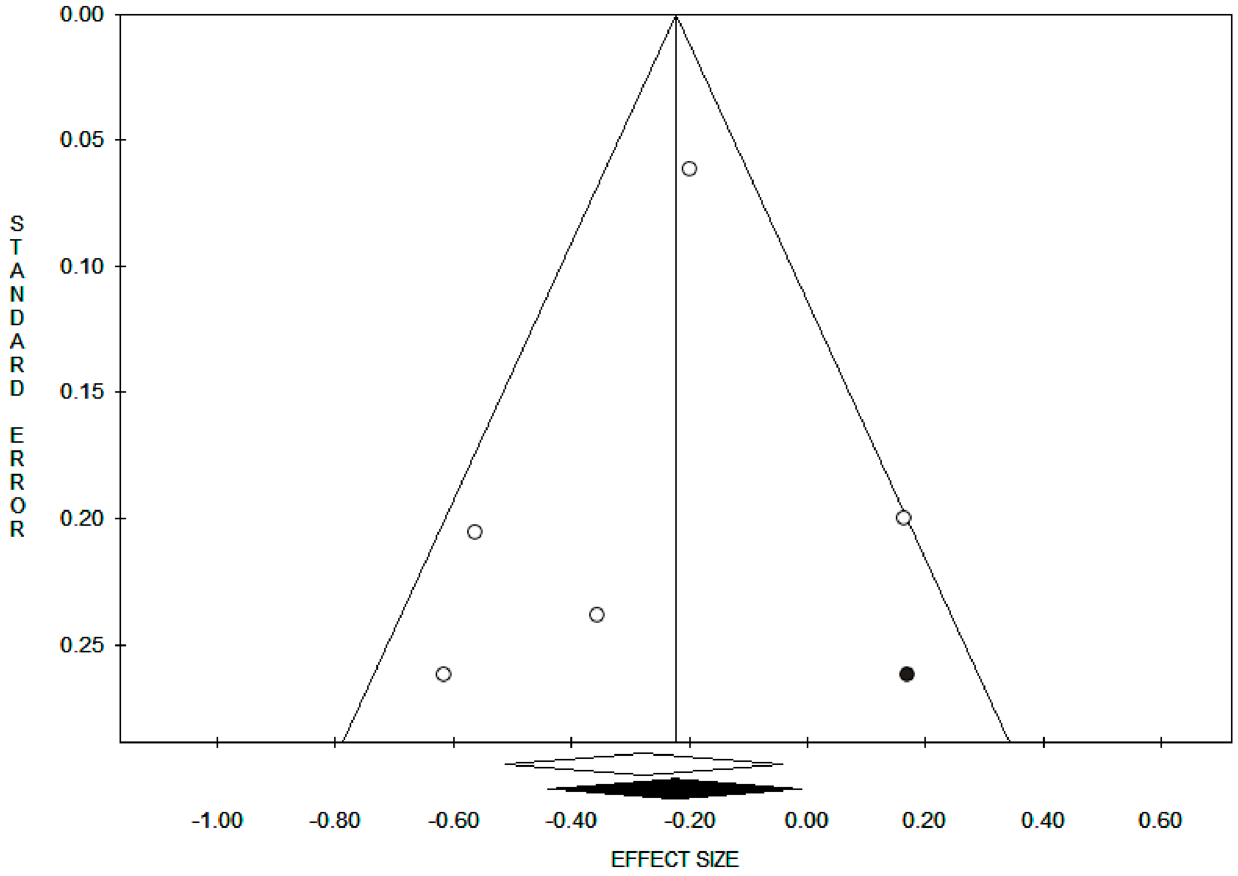

Supplement: Supplementary file 1 [file ijerph-20-02403-s001.zip › Supplementary.pdf]
